# Corncob as an effective, eco-friendly, and economic biosorbent for removing the azo dye Direct Yellow 27 from aqueous solutions

**DOI:** 10.1371/journal.pone.0196428

**Published:** 2018-04-26

**Authors:** Nayda Karina Berber-Villamar, Alma Rosa Netzahuatl-Muñoz, Liliana Morales-Barrera, Griselda Ma. Chávez-Camarillo, César Mateo Flores-Ortiz, Eliseo Cristiani-Urbina

**Affiliations:** 1 Instituto Politécnico Nacional, Escuela Nacional de Ciencias Biológicas, Departamento de Ingeniería Bioquímica, Unidad Profesional Adolfo López Mateos, Delegación Gustavo A. Madero, Ciudad de México, México; 2 Universidad Politécnica de Tlaxcala, Colonia San Pedro Xalcaltzinco, Tepeyanco, Tlaxcala, México; 3 Unidad de Biotecnología y Prototipos, Facultad de Estudios Superiores-Iztacala, Universidad Nacional Autónoma de México, Los Reyes Iztacala, Tlalnepantla, Estado de México, México; 4 Laboratorio Nacional en Salud, Facultad de Estudios Superiores-Iztacala, Universidad Nacional Autónoma de México, Los Reyes Iztacala, Tlalnepantla, Estado de México, México; VIT University, INDIA

## Abstract

The corncob is an agricultural waste generated in huge quantities during corn processing. In this paper, we tested the capacity of corncob particles for water purification by removing the azo dye Direct Yellow 27 (DY27) via biosorption. The biosorption process was investigated in terms of the kinetics, equilibria, and thermodynamics. Batch biosorption studies showed that the biosorption performance has strong inverse correlations to the solution pH and the corncob particle size, and it increases quickly with increasing contact time and initial dye concentration. The pseudo-second-order kinetic model provides the best fit to the experimental data, whereas the Redlich-Peterson isotherm model is most suitable for describing the observed equilibrium biosorption. The biosorption process is exothermic, spontaneous, and physisorption in character. Fourier transform infrared (FTIR) spectroscopy and confocal scanning laser microscopy (CSLM) studies suggest that lignocellulose and proteins play key roles in the biosorption of DY27 from aqueous solutions by corncob. Furthermore, after biosorption onto the corncob, the dye can be effectively desorbed using 0.1 M NaOH solution. Therefore, the corncob can be used as a promising biosorbent to remediate DY27-contaminated water and wastewater.

## Introduction

Synthetic organic dyes are extensively used in many technologies, such as textiles, printing, paper making, leather tanning, rubber, food processing, plastics, cosmetics, hair coloring, pharmaceuticals, photography, agricultural research, light-harvesting arrays, and photoelectrochemical cells [1–3]. Currently, there are estimated to be more than 100,000 commercially available dyes with a total production rate of more than 0.7–1 × 10^6^ tons per year [1].

Approximately 12% of the synthetic dyes produced annually in the world are believed to be lost during manufacturing and processing operations, and about 20% of the lost dyes enter industrial wastewaters [4]. Such wastewaters are often discharged directly into natural water bodies without treatment. This has caused considerable adverse impacts on the environment and human health [2, 5]. For example, even a very small amount of dye (less than 1 mg L^-1^) can markedly color the water, making it unfit for human consumption [6, 7]. Such colored water also has reduced sunlight penetration, which lowers photosynthetic activities in the water and increases the heterotrophic activity that depletes dissolved oxygen [8]. Furthermore, dyes may also cause eutrophication; prevent re-oxygenation of water bodies; pollute ground water; and be toxic to aquatic plants, microorganisms, fish, and mammals [9]. Especially, the impurities and (bio)transformation products of certain synthetic dyes directly affect human health due to their acute and/or chronic toxicity; allergenic, mutagenic, carcinogenic, genotoxic, cytotoxic, estrogenic, and anti-estrogenic activities; and immune suppression effects [9–12].

Many chemical, physical, and biological technologies have been developed to remove dyes from aqueous solutions. They include: coagulation-flocculation, precipitation-flocculation, electrokinetic coagulation, electrocoagulation-electroflotation, photocatalysis, oxidation, advanced oxidation, electrochemical treatment, adsorption on activated carbon, membrane separation, ion exchange, irradiation, incineration, biodegradation, and decolorization by bacterial, white-rot fungal, or mixed cultures [1, 7, 13]. However, each of these technologies has drawbacks, such as operational problems, high cost, and the generation of secondary wastes that could be even more toxic and/or difficult and expensive to remove/degrade [14–16]. In comparison, biosorption has been considered a promising technology for the removal of dyes from aqueous solutions, owing to its higher treatment efficiency and efficacy, faster removal rate, high flexibility, and low capital investment and operating costs. This method is also easy to design, implement, and operate. Furthermore, being an environmentally friendly method, it minimizes secondary waste production and allows possible dye recovery and biosorbent regeneration afterwards [15, 17].

Agricultural wastes have attracted significant attention for biosorption of dyes from aqueous solutions. Many of these materials display excellent performance, low or zero cost, natural abundance, high availability, eco-friendliness, and renewability. The functional groups on their surface could bind to many classes of dyes with selectivity [1, 6, 18]. Corn is one of the most widely planted crops in the world. During the processing of corn, a large volume of corncobs is generated as agricultural waste [19, 20]. The amount of corncobs generated worldwide is approximately 144 million tons per year [21], and most of it is discarded or burnt, causing serious environmental pollution and no benefit [22, 23]. Considering this, corncobs may be used as a value-added biosorbent for the removal of toxic organic and inorganic pollutants from wastewaters [22, 24].

In the present work, we assess the potential of corncobs to remove the Direct Yellow 27 (DY27), which is a monoazo direct dye with good light fastness [25]. DY27 is extensively used for the dyeing and printing of paper, leather, as well as of cotton, nylon, rayon, and viscose fabrics [3]. Consequently, DY27 has been found in many industrial wastes in aqueous environments [26]. The biosorbent made from corncobs was characterized by its chemical composition, pH at the point of zero charge (pH_pzc_), Fourier transform infrared (FTIR) spectroscopy, scanning electron microscopy (SEM), and confocal scanning laser microscopy (CSLM). The influence of different process parameters (such as solution pH, corncob particle size, shaking contact time, and initial DY27 concentration) on the kinetics of DY27 biosorption from aqueous solutions by corncob was studied. Additionally, the isotherms and thermodynamics of DY27 biosorption were examined. Finally, the optimal elute for DY27 desorption from the used biosorbent and the associated kinetics were also discussed. It is worth it to mention that no previous works have addressed the biosorption of DY27 from aqueous solution.

## Materials and methods

### Ethics statement

No specific permits were required for the corncob sample collection for this study. We used corncob material that came from the private lands from one of the authors (NKBV). No material was taken from protected land or National Parks. The studies conducted in this work did not involve any endangered or protected species.

### Chemicals

**Fig 1:**
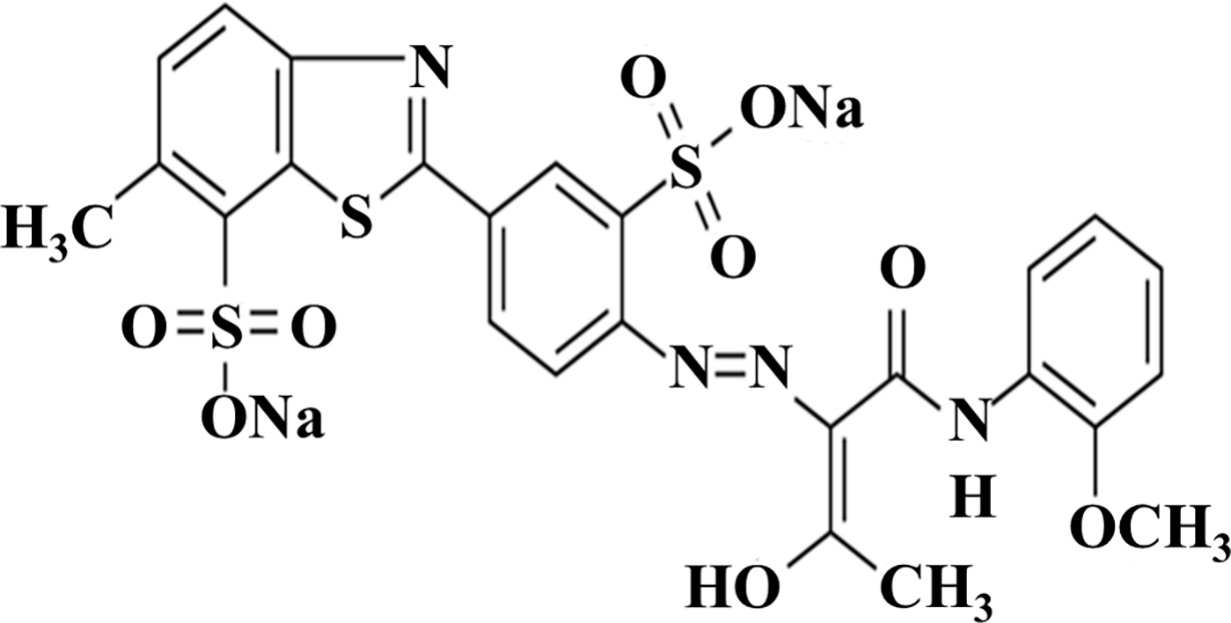
Chemical structure of Direct Yellow 27.

DY27 dye (molecular formula: C_25_H_20_N_4_Na_2_O_9_S_3_, molecular weight: 662.62 g mol^-1^, Fig 1) [27] was purchased from Sigma-Aldrich Chemicals. All other reagents were of analytical grade. A DY27 stock solution of 2 g L^-1^ was prepared by dissolving an accurately weighed amount of DY27 in distilled deionized water. Test solutions of DY27 were prepared by diluting the stock solution with distilled deionized water. In this work, the initial DY27 concentration (*C*_*o*_) varied from 7 to 293 mg L^-1^, and the pH of each solution was adjusted to the desired value using 0.1 M HCl or NaOH solutions.

### Biosorbent preparation

Corncobs (*Zea mays* L.) were collected in the municipality of Villa del Carbón (Latitude: 19° 43’ 41”N; Longitude: 99° 28’ 28”W), State of Mexico, Mexico. The collected corncobs were first washed thoroughly with distilled deionized water, and then oven-dried at 60°C until the weight was constant. The dried samples were subsequently ground in a hammer mill (Glen Creston Ltd.), and the resulting particles were screened using ASTM standard sieves to obtain fractions with different particle sizes. The sieved fractions were stored in airtight plastic containers until use.

### Proximate chemical composition of corncob

The biosorbent's proximate chemical composition was analyzed in triplicate, following methods described by the Association of Official Analytical Chemists [28]. The total protein (*TP*) was analyzed by the Kjeldahl method, using 6.25 as the conversion factor from total nitrogen to total protein. Ash (*A*) was determined by incineration of samples in a muffle furnace at 600°C until the weight was constant. Ether extract (*EE*) was determined by the Soxhlet method. Crude fiber (*CF*) was measured as the loss on ignition of dried lipid-free residues after digestion with H_2_SO_4_ and NaOH standard solutions. Nitrogen-free extract (*NFE*) was calculated as the difference between dry matter and the sum of total protein, ether extract, ash, and crude fiber (Eq 1):

%NFE=100−%A−%TP−%EE−%CF (1)

### Determination of point of zero charge

The biosorbent's point of zero charge (pH_pzc_) is defined as the pH at which the amounts of positive and negative charges on its biosorbent surface are equal, leading to a neutral net charge [29]. In the present work, pH_pzc_ was determined using a batch equilibrium method, following the procedures described by Lim et al. [30]. Briefly, corncob samples (1 g L^-1^) were mixed with 0.1 M KNO_3_ solutions which were previously adjusted to initial pH (pH_i_) values of 2, 4, 6, 8, 10, and 12 by the addition of 0.1 M HNO_3_ and/or 0.1 M NaOH. The suspensions were agitated continuously at 150 rpm at 18 ± 1°C for 48 h to ensure that an equilibrium pH was reached in each case. Subsequently, the suspensions were filtered through filter paper (Whatman, grade 42), and the final pH (pH_f_) of each filtrate was recorded. A graph of the pH change in the KNO_3_ solution (ΔpH = pH_i_−pH_f_) against pH_i_ was then constructed to determine the pH_pzc_ of corncob.

### Batch kinetic experiments for DY27 biosorption

Batch kinetic biosorption studies were conducted to evaluate the effect of relevant operational parameters, such as solution pH, corncob particle size, shaking contact time, and *C*_*o*_ on the biosorption of DY27 by corncob. All experiments were performed in 500-mL Erlenmeyer flasks, using 100 mL of DY27 solution of known pH and dye concentration and 1 g (dry weight) L^-1^ of biosorbent. During each experiment, the pH of the solution was maintained at a constant value (± 0.1 pH unit) by periodic checks and adjustments with 0.1 M HCl or NaOH solution. The flasks were placed in a shaker bath (Cole Parmer®) at a constant shaking speed of 150 rpm for 72 h.

The influence of solution pH was examined at *C*_*o*_ = 50 mg L^-1^ at different pH values (1.5, 2.0, 2.5, 3.0, 4.0, 5.0, and 6.0) at 18 ± 1°C. The effect of particle size was explored in DY27 solution at *C*_*o*_ = 50 mg L^-1^, pH 1.5, and with different corncob particle sizes (0.297–0.50, 0.50–0.80, 0.8–1.0, 1.0–1.18, 1.18–1.41, 1.41–1.70, and 1.70–2.0 mm) at 18 ± 1°C. The influence of shaking contact time was investigated for 0, 0.1, 0.25, 0.5, 0.75, 1.0, 1.5, 2.0, 2.5, 3.0, 4.0, 5.0, 6.0, 7.0, 24, 48, and 72 h in solutions with different initial dye concentrations (7, 13, 28, 50, 74, 85, 100, 145, and 293 mg L^-1^) at pH 1.5 and 18 ± 1°C. These *C*_*o*_ values were also used to examine the kinetic biosorption profiles at pH 1.5 and 18 ± 1°C.

Corncob-free controls were used to evaluate the loss of DY27 dye arising from precipitation and/or adsorption onto glass wall.

For the kinetic studies, after certain contact times, samples were taken from the mixture and centrifuged at 5000 rpm for 10 min. The DY27 concentration in the supernatants was analyzed by spectrophotometry (Thermo Scientific™ Evolution 201) at 398 nm. The time-dependent DY27 biosorption capacity (*q*_*t*_, mg g^-1^) was estimated using the following mass balance relationship:

$$q_{t}=\frac{\left(C_{0}-C_{t}\right)}{M}$$ (2)

Here, *C*_*o*_ and *C*_*t*_ are the initial and residual DY27 concentrations (mg L^-1^) at time *t* = 0 and *t* (h), respectively, and *M* is the corncob concentration (g L^-1^).

### Isotherm studies of DY27 biosorption

For DY27 equilibrium biosorption, corncob biomass was added at 1 g (dry weight) L^-1^ to DY27 solutions at pH = 1.5 at *C*_*o*_ = 7–293 mg L^-1^ and different temperatures (18, 35, 50, and 60°C), with a constant agitation of 150 rpm for 72 h to ensure that biosorption equilibrium was reached. Afterwards, samples were taken, centrifuged at 5000 rpm for 10 min, and then the dye concentrations in the supernatants were measured. The DY27 equilibrium biosorption capacity (*q*_*e exp*_) was calculated using Eq (2), by replacing *C*_*t*_ with *C*_*e*_ (the equilibrium concentration of DY27 in the aqueous solution in mg L^-1^).

### Kinetic modeling of DY27 biosorption

Modeling the kinetics of target pollutant removal is crucial for understanding relevant aspects of the biosorption process, such as the biosorption rate and its influencing factors, designing effective and efficient biosorption systems, selecting operation conditions for full-scale batch process, and establishing the time dependence of biosorption systems under different process conditions [31, 32]. In the present work, the dynamics of DY27 biosorption onto corncob biomass was examined at different solution pH levels, corncob particle sizes, and *C*_*o*_. The data were analyzed using five different kinetic models: pseudo-first-order, pseudo-second-order, Elovich, intraparticle diffusion, and fractional power (Table 1).

**Table 1:** Different kinetic, isotherm, and thermodynamic models.

**Kinetic models**			
	**Equation**	**Nomenclature**	**Reference**
**Pseudo-first-order**	$\log\;\left(q_{e1}-q_{t}\right)=\log q_{e1}-\frac{k_{1}}{2.303}t$	*q*_*e*1_ *and q*_*t*_: biosorption capacity [mg g^-1^] at equilibrium and at any time *t* [h], respectively; *k*_1_: rate constant of the model [h^-1^]	[33]
**Pseudo-second-order**	$\frac{t}{q_{t}}=\frac{1}{k_{2}q_{e2}^{2}}+\frac{1}{q_{e2}}t$	*q*_*e*2_ *and q*_*t*_: biosorption capacity [mg g^-1^] at equilibrium and at any time *t* [h], respectively; *k*_2_: rate constant of the model [h^-1^]	[34]
**Intraparticle diffusion**	*q*_*t*_ = *k*_*id*_*t*^0.5^ + *c*	*q*_*t*_: biosorption capacity [mg g^-1^] at any time *t* [h]; *k*_*id*_: intraparticle diffusion rate constant [mg g^-1^ h^-0.5^]; *c*: model intercept	[35]
**Elovich**	$q_{t}={\frac{1}{B_{E}}ln(A_{E}B_{E})+\frac{1}{B}}_{E}ln\;t$	*A*_*E*_: initial biosorption rate [mg g^-1^ h ^-1^]; *B*_*E*_: desorption constant of the model [g mg^-1^]	[16]
**Fractional power**	*q*_*t*_ = *k*_*fp*_*t*^*v*^	*v*: fractional power rate constant [h^-1^]; *k*_*fp*_: fractional power model constant [mg g^-1^]	[16]
**Isotherm models**			
**Two-parameter models**			
**Langmuir**	$q_{e}=\frac{q_{m}K_{L}C_{e}}{1+K_{L}C_{e}}$	*q*_*e*_ *and q*_*m*_: biosorption capacity at equilibrium and maximum biosorption capacity [mg g^-1^], respectively; *C*_*e*_: liquid phase concentration of dye at equilibrium [mg L^-1^]; *K*_*L*_: Langmuir constant [L mg^-1^]	[30]
**Freundlich**	$q_{e}=K_{F}{C_{e}}^{\frac{1}{n_{F}}}$	*K*_*F*_: constant of the Freundlich model [(mg g^-1^)(mg L^-1^)^-1/nF^]; *n*_*F*_: heterogeneity factor.	[16]
**Temkin**	$q_{e}=\frac{RT}{B_{T}}ln(A_{T}C_{E})$	*T*: absolute temperature [K]; *R*: ideal gas constant [8.314 J mol^-1^ K^-1^]; *B*_*T*_: constant related to heat of biosorption [J mol^-1^]; *A*_*T*_: Temkin isotherm constant [L mg^-1^]	[35]
**Halsey**	$q_{e}={\left(K_{H}/C_{e}\right)}^{1/n_{H}}$	*K*_*H*_: Halsey isotherm model constant [L g^-1^]^-1/nH^; *n*_*H*_: Halsey model exponent	[16]
**Dubinin-Radushkevich**	$q_{e}=q_{m}exp(-B_{DR}E_{DR}^{2})$	*B*_*DR*_: biosorption energy constant [mol^2^ J^-2^]; *E*_*DR*_: Polanyi potential [kJ mol^-1^]	[36]
**Three-parameter models**			
**Sips**	$q_{e}=q_{m}\frac{{\left(K_{S}C_{e}\right)}^{1/n_{s}}}{1+{\left(K_{S}C_{e}\right)}^{1/n_{s}}}$	*K*_*s*_: Sips constant; 1/*n*_*s*_: Sips model exponent	[30]
**Toth**	$q_{e}=\frac{q_{m}b_{T}Ce}{{\left(1+{(b}_{T}{Ce)}^{{n_{T}}^{-1}}\right)}^{n_{T}}}$	*b*_*T*_: Toth model constant [L mg^-1^]^-1/nT^; *n*_*T*_: Toth model exponent	[36]
**Redlich-Peterson**	$q_{e}=\frac{K_{RP}C_{e}}{1+A_{RP}C_{e}^{B_{RP}}}$	*K*_*RP*_: Redlich-Peterson model isotherm constant [L g^-1^]; *A*_*RP*_: Redlich-Peterson model constant [L mg^-1^]^BRP^; *B*_*RP*_: Redlich-Peterson model exponent	[36]
**Radke-Prausnitz**	$q_{e}=\frac{A_{R}R_{R}C_{e}^{B_{R}}}{A_{R}{+R}_{R}C_{e}^{B_{R-1}}}$	*A*_*R*_: [L g^-1^] and *R*_*R*_ [L mg^-1^]: Radke-Prausnitz model constants; *B*_*R*_: Radke-Prausnitz model exponent	[16]
**Thermodynamic models**			
**Gibbs free energy change**	Δ ***G***° = −*R T* ln *K*_*c*_ $K_{C}=\frac{q_{e}}{C_{e}}$	*T*: absolute temperature [K]; *R*: ideal gas constant [8.314 J mol^-1^ K^-1^]; *K*_*c*_: equilibrium constant [L g^-1^]; Δ ***G***°: Gibbs free energy change [J mol^-1^]	[37]
**Entropy change**	∂Δ***G***° = ∂*T* ∂Δ***S***°	*T*: absolute temperature [K]; Δ ***S***°: entropy change [J mol^-1^ K^-1^]	[37]
**Enthalpy change**	Δ ***H***° = Δ***G***° − *T* Δ***S***°	Δ ***H***°: enthalpy change [J mol^-1^]	[16]

### Modeling of DY27 biosorption equilibrium isotherms

Equilibrium biosorption isotherm models are useful for describing the interactions between the adsorbate and biosorbent, and therefore they are essential for the design of biosorption facilities on a large scale [32]. Various two-parameter (Langmuir, Freundlich, Temkin, Halsey, and Dubinin-Radushkevich) and three-parameter (Sips, Toth, Radke-Prausnitz, and Redlich-Peterson) isotherm models (Table 1) were applied in the present work to fit the experimental equilibrium data of DY27 biosorption.

### Thermodynamic analysis

The Gibbs free energy change (*ΔG*^*o*^, J mol^-1^), enthalpy change (*ΔH*^*o*^, J mol^-1^), and entropy change (*ΔS*^*o*^, J mol^-1^ K^-1^) were calculated in order to describe the thermodynamic behavior of DY27 biosorption onto corncob biomass (Table 1).

### Desorption studies

Corncob samples were mixed with a 293 mg L^-1^ DY27 solution (100 mL) at a ratio of 1 g L^-1^, pH 1.5, and 18 ± 1°C with constant agitation at 150 rpm for 72 h to ensure biosorption equilibrium. Afterwards, the DY27-loaded sample was separated from the dye solution by centrifugation at 5000 rpm for 10 min, and the concentration of residual DY27 in the supernatant was measured to calculate the amount of dye biosorption. The DY27-loaded precipitate was gently washed with distilled deionized water to remove any unbiosorbed dye. After another centrifugal separation, the dye-loaded corncob was oven-dried at 60°C until a constant dry weight was obtained, and stored in an airtight plastic container until use.

All batch desorption experiments were conducted in 500-mL Erlenmeyer flasks containing 100 mL of given desorption solution and 1 g (dry weight) L^-1^ of DY27-loaded corncob. The flasks were agitated in a shaker bath (Cole-Parmer®) at 150 rpm and 18 ± 1°C.

To select the best desorption solutions, different acids (0.1 M HCl, H_2_SO_4_, HNO_3_, NH_4_Cl, and (NH_4_)_2_SO_4_), alkali (0.1 M NaOH, KOH, and Na_2_CO_3_), neutral salts (0.1 M NaCl and NaNO_3_), and organic compounds (0.1 M acetone and chloroform) were mixed with DY27-loaded corncob over a 48 h period. The best agent for DY27 desorption was used for batch desorption kinetic studies. The amount of desorbed DY27 was determined spectrophotometrically at a wavelength of 398 nm. The desorption efficiency (*E*_*Dt*_, %) was estimated according to the following equation:

$$E_{Dt}=\frac{C_{Dt}}{q_{e}M}\times 100$$ (3)

Here, *C*_*Dt*_ is the residual DY27 concentration (mg L^-1^) in the desorption solution after time *t*_*D*_, and *q*_*e*_ is the initial (*t*_*D*_ = 0 h) dye concentration in the corncob (mg g^-1^), i.e., the equilibrium biosorption capacity.

### Statistical data analysis

All biosorption and desorption experiments were repeated three times, and the values reported herein represent the average values. The biosorption and desorption data were statistically analyzed by analysis of variance (ANOVA) with Tukey's test (overall confidence level = 0.05) using GraphPad Prism software (version 7.0; GraphPad Software, Inc.). The same software was also used to estimate all the biosorption model parameters by nonlinear regression analysis. The different biosorption models used for fitting were assessed using the coefficient of determination (*r*^*2*^), standard deviation of residuals (*Sy*.*x*), absolute sum of squares (*ASS*), and Aikake information criterion (*AIC*). A high value of *r*^*2*^ (close to 1.0) and small *Sy*.*x*, *ASS*, and *AIC* values correspond to better representation of experimental data by a particular model.

### SEM analysis

To analyze the morphological features and surface characteristics of native (i.e. not loaded with DY27) and DY27-loaded corncob samples, micrographs were obtained using a high-resolution scanning electron microscope (JSM-7800F, JEOL) at an accelerating voltage of 5 kV after gold coating.

### CSLM analysis

DY27-loaded corncob samples were stained with 1% fluorescein or calcofluor white M2R (Sigma-Aldrich) in order to detect proteins and cellulose, respectively. Excess dye was removed with deionized water, and the samples were dried at 60°C for 24 h. Micrographs were obtained using a multiphoton laser scanning confocal microscope (LSM710 NLO, Carl Zeiss) equipped with an argon/krypton laser with four excitation lines (405, 488, 561, and 633 nm) and powers of 100, 100, 2, and 2%. All images were acquired at 10× magnification without zooming, and saved in TIFF format at 512 × 512 pixels.

### FTIR analysis

FTIR spectroscopic analysis of native and DY27-loaded corncob samples was used to identify the main functional groups on the corncob surface that may be involved in the biosorption of DY27 from aqueous solutions. For this purpose, finely ground native or DY27-loaded corncob were thoroughly mixed with dried spectroscopic KBr in a 1:3 ratio, and immediately analyzed using a Thermo Scientific Nicolet iS10 FTIR spectrometer equipped with an EasiDiff diffuse reflectance hemisphere accessory (Pike Technologies). FTIR spectra were obtained using 64 scans over the range of 4000–400 cm^-1^ with a resolution of 1 cm^-1^. The Kubelka-Munk function was calculated from the diffuse reflectance data using the OMNIC software (Thermo Fischer Scientific). The FTIR spectra in the present work are presented in Kubelka-Munk function vs. wavenumber.

## Results and discussion

### Chemical composition of corncobs

The proximate chemical composition of corncobs (dry matter basis) was found to be: total protein, 4.28 ± 0.63%; total ash, 3.44 ± 0.08%; ether extract, 2.03 ± 0.22%; crude fiber, 26.29 ± 0.52%; and nitrogen-free extract, 63.96 ± 1.5%. Therefore, the corncob has low fat, protein, and mineral contents, and rich in carbohydrate and lignin contents. This is consistent with the knowledge that the main components of corncobs are cellulose, hemicellulose, and lignin, which together comprise approximately 90% of the dry matter [20, 21]. The polymeric components in corncob possess many functional groups that can be active sites for the biosorption of dyes [20].

### pH_pzc_ of corncob

**Fig 2:**
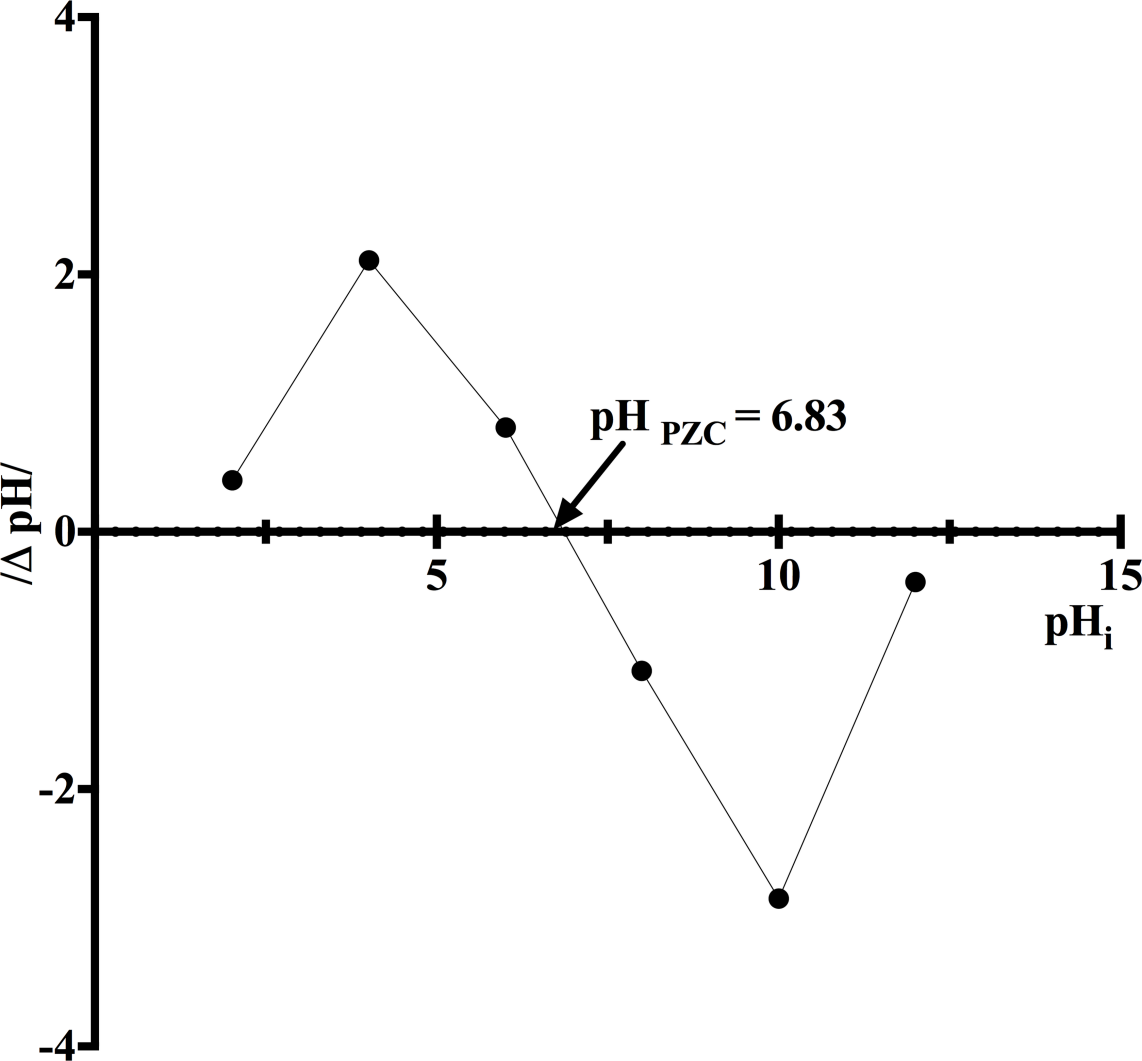
Determination of point of zero charge (pH_pzc_) of corncob.

The solution pH strongly affects the surface charge of a biosorbent and its biosorption capacity. The surface charge can be qualitatively assessed by the point of zero charge (pH_pzc_) or isoelectric point [2, 23]. The pH_pzc_ of corncob was determined to be 6.83 (Fig 2). Therefore, for solution pH below this value, the corncob particles carry positive charges, and vice versa. The value of 6.83 is close to that reported by Leyva-Ramos et al. [38] (pH_pzc_ = 6.2), who attributed the weak acidity of natural corncob to the slightly higher concentration of acid sites than that of the basic ones.

### SEM analysis

**Fig 3:**
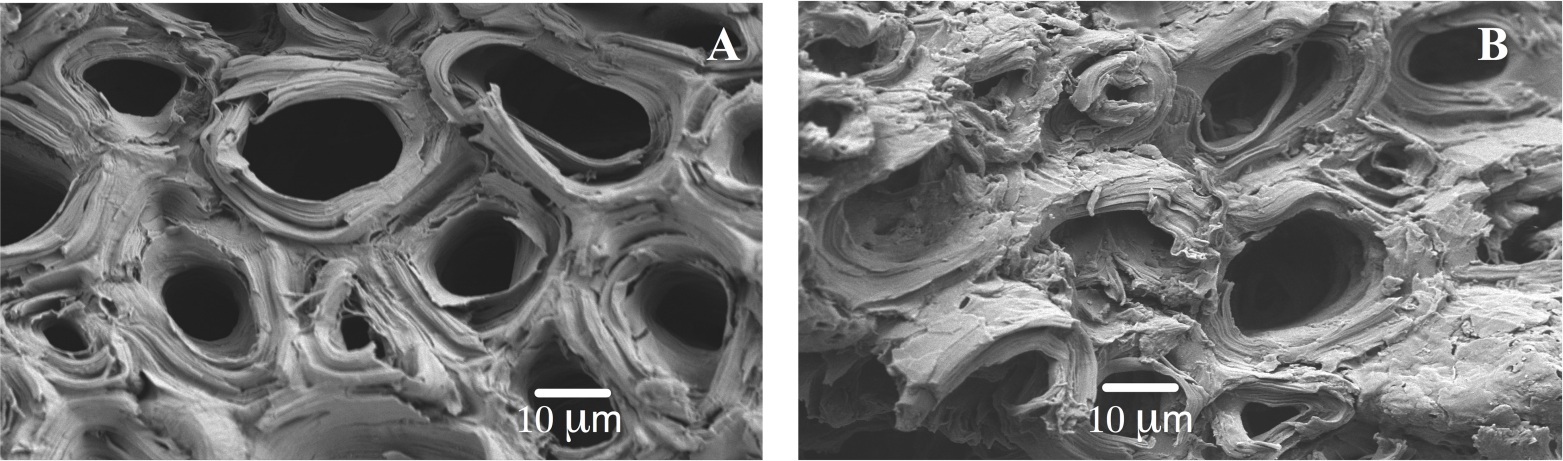
SEM micrographs of native (A) and DY27-loaded (B) corncob at 1200× magnification.

SEM has been widely used to directly observe the surface structure and morphology of different biosorbents [18]. Fig 3 shows the SEM micrographs of DY27-unloaded (native) and DY27-loaded corncob. SEM micrographs revealed that native corncob has a rough and porous surface (Fig 3A), with pores of different shapes and with sizes exceeding 50 nm, thus indicating that corncob has a macroporous structure. Likewise, corncob has an irregular structure, which makes biosorption of DY27 dye on different parts of the biosorbent possible. From the SEM images of the native (Fig 3A) and DY27-loaded corncob (Fig 3B), there is no evidence of changes in the surface morphology of the biosorbents. Contrastingly, some studies have mentioned that the surface morphology of biosorbents may change once dyes have been loaded [18, 39].

### Effect of solution pH on DY27 biosorption kinetics

No measurable changes in the DY27 dye concentration were detected in any of the assayed corncob-free controls, thus indicating that any evident DY27 removal was solely due to the biosorbent.

As mentioned earlier, the pH can affect the biosorption of dyes from aqueous solutions via changing the surface charge of biosorbents. The solution pH may also affect the kinetics of biosorption through the ionization state and solubility of dyes [2].

**Fig 4:**
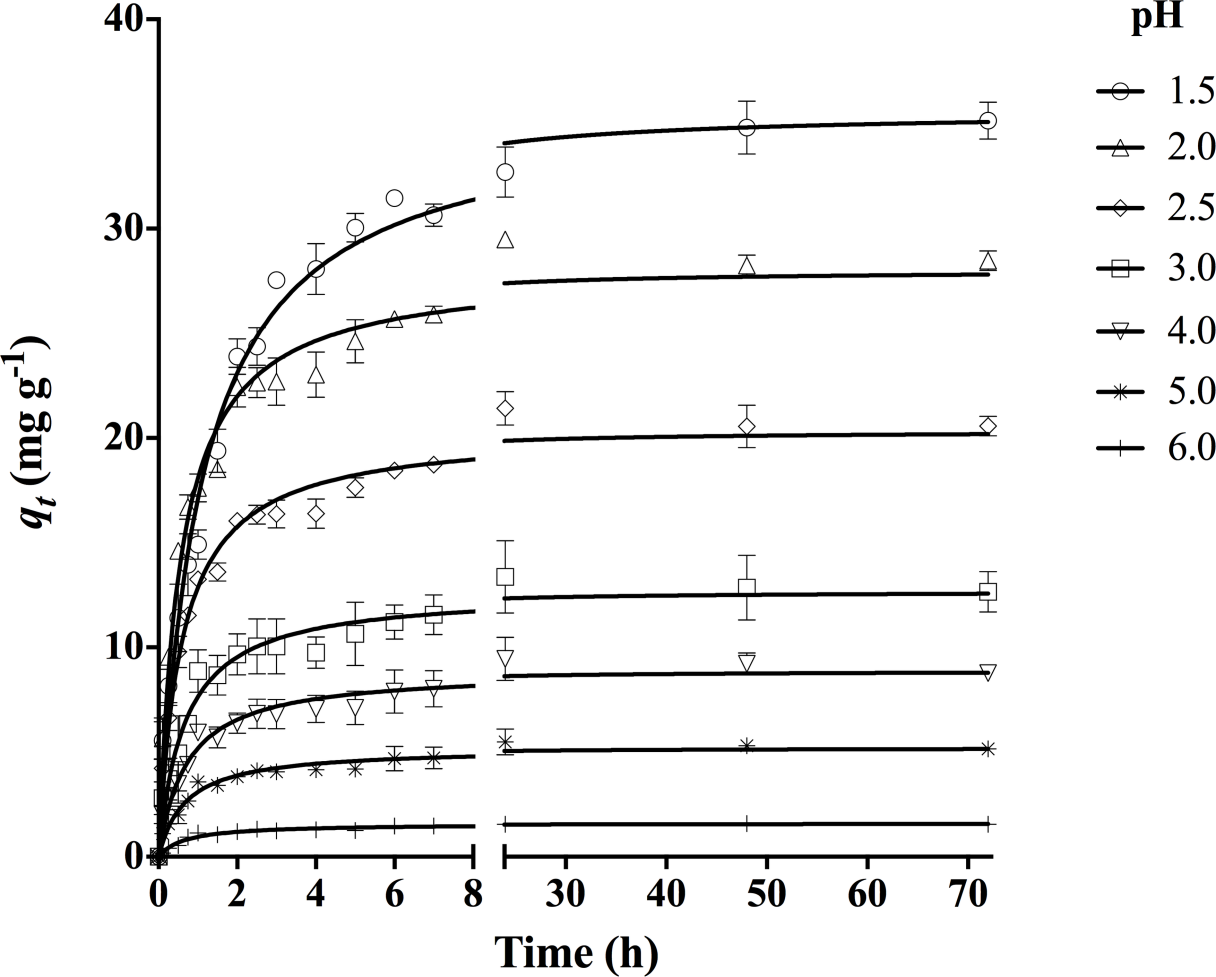
Influence of solution pH on DY27 biosorption capacity of corncob.

Fig 4 depicts the kinetic profiles of DY27 biosorption by corncob at different solution pH (1.5–6.0). Solutions with pH < 1.5 could not be assayed because of dye precipitation. Corncob was found to take up DY27 from aqueous solution at all other pH levels, although the rate and extent of biosorption varied greatly. The capacity of DY27 biosorption increased as the solution pH decreased, reaching its highest level at pH 1.5. This trend can be explained with the aid of pH_pzc_. All the DY27 solutions assayed had pH < pH_pzc_. Under these conditions, the surface charge of corncob is positive because the active biosorption binding sites are protonated. Such protonation favors the biosorption of anionic DY27 molecules as a result of electrostatic attraction. The lower the solution pH, the stronger the corncob surface is protonated and the greater the biosorption of DY27.

The present results clearly show that the optimal solution pH for the biosorption of DY27 onto corncob is 1.5, and this pH was therefore used in all further studies. To the best of our knowledge, no previous works have addressed the biosorption of DY27 from aqueous solution. Only a single study reported the removal of DY27 from aqueous solution using an inorganic adsorbent (crystalline hydroxyapatite), for which the optimal pH for adsorption was found to be 3.5 (the lowest pH value assayed by those authors) [26].

### Influence of corncob particle size on DY27 biosorption

**Fig 5:**
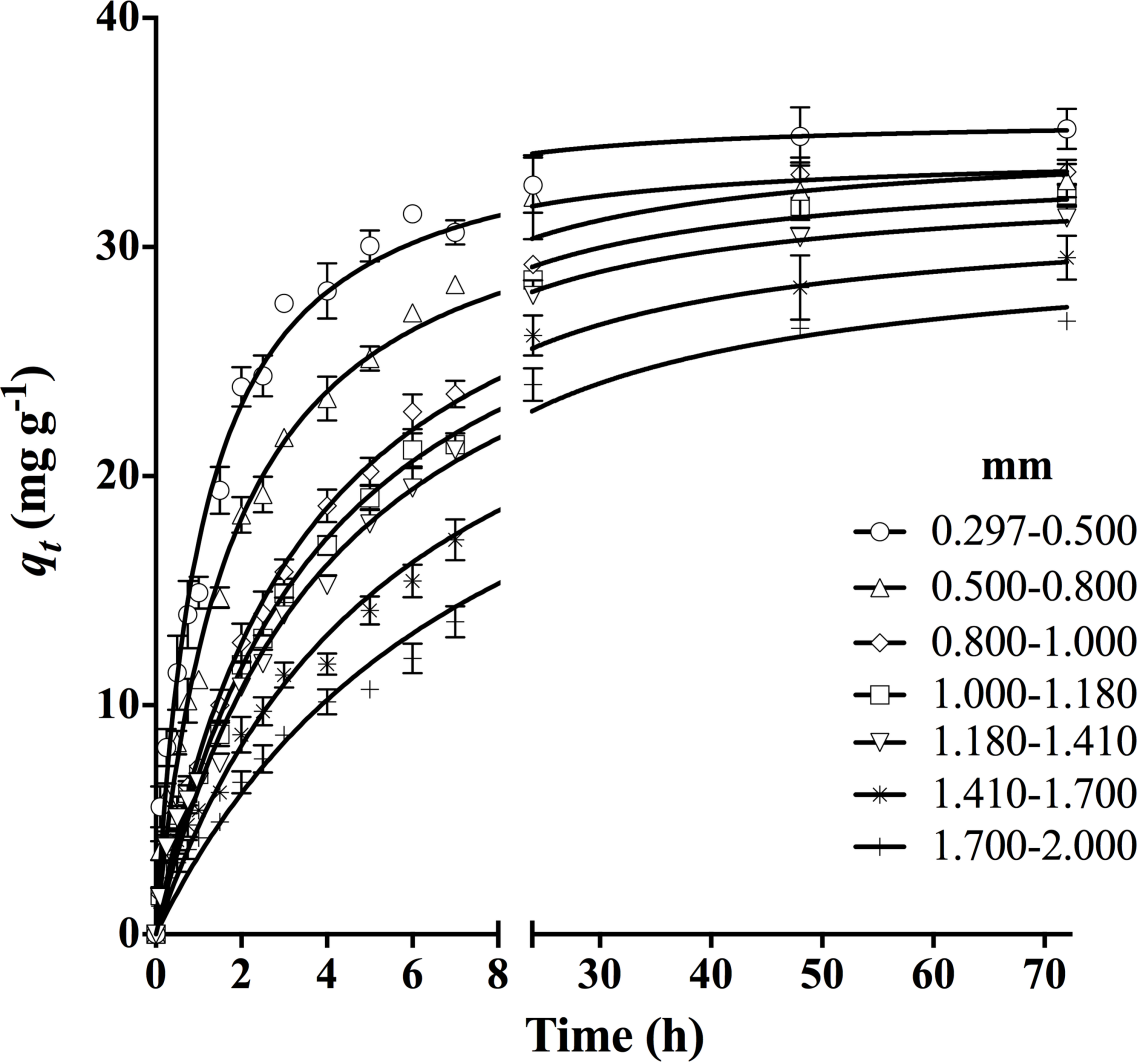
Effect of corncob particle size on DY27 biosorption capacity of corncob.

Fig 5 displays the effects of corncob particle size on DY27 biosorption at pH 1.5. It is apparent that the rate and capacity of biosorption increased for finer corncob particles. This trend is attributed to the enlarged specific surface area, as well as a lower intraparticle diffusion resistance that improves accessibility of internal sites on the corncob [1, 40, 41]. Based on this result, the smallest particle size (0.297–0.50 mm) was used in further studies.

### Effect of contact time and initial DY27 concentration on biosorption

**Fig 6:**
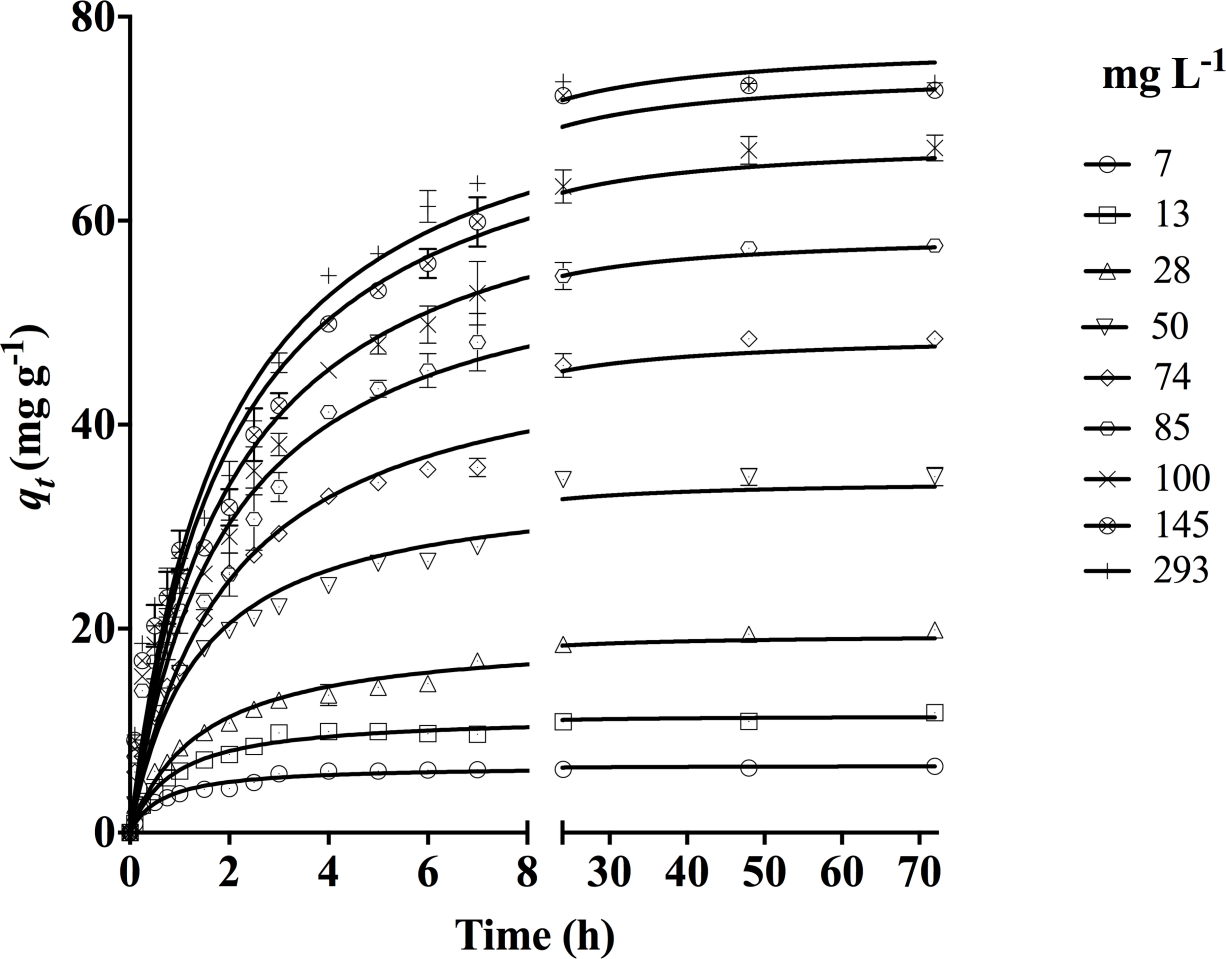
Influence of initial DY27 concentration on its biosorption capacity of corncob.

From the results shown in Fig 6, it is evident that the contact time between biosorbent and DY27 solution has a significant effect on the biosorption. At all assayed *C*_*o*_ values, the biosorption capacity gradually increased with the experiment time, eventually reaching a maximum constant value that corresponded to the equilibrium biosorption capacity (*q*_*e exp*_). After this point, the DY27 removal ceased. Furthermore, irrespective of *C*_*o*_, the initial biosorption rate was quite fast because (1) there were a large number of positively charged binding sites available for biosorption and (2) the driving force to transfer DY27 molecules from the aqueous solution to the surface of corncob was high [2, 30]. Afterwards, both the number of vacant binding sites and the mass transfer driving force decreased gradually, therefore the biosorption rate decreased and eventually reached dynamic equilibrium [1, 42].

Fig 6 also shows that the DY27 equilibrium biosorption capacity increased from 6.5 to 73.00 mg g^-1^ as *C*_*o*_ was increased from 7 to 145 mg L^-1^. The higher availability of DY27 molecules in the solution increased their interactions with the corncob and the equilibrium biosorption capacity. A higher *C*_*o*_ also increases the DY27 concentration gradient near the particle surface, which is the thermodynamic driving force for the mass transfer of DY27 from the solution to the corncob surface. Consequently, the probability of interaction between the dye molecules and the active biosorption sites increases, and so does the DY27 biosorption capacity [16]. However, the equilibrium biosorption capacities were very similar at *C*_*o*_ = 145 and 293 mg L^-1^, probably due to saturation of the biosorption sites.

The above results clearly indicate that there is a direct relation between the dye concentration and the available biosorption sites on the corncob surface, the latter is the limiting factor for DY27 biosorption, and that the saturation of biosorbent depends strongly on *C*_*o*_ [1, 16, 18].

### Modeling of biosorption kinetics

The kinetics of DY27 biosorption onto corncob was analyzed using five different models at pH = 1.5–6.0, corncob particle size from 0.297–0.5 to 1.7–2.0 mm, and *C*_*o*_ = 7–293 mg L^-1^. Tables 2–4 present the experimental equilibrium biosorption capacity (*q*_*e exp*_), the kinetic parameter values (pseudo-first-order model: *q*_*e1*_ and *k*_*1*_, pseudo-second-order model: *q*_*e2*_ and *k*_*2*_, Elovich model: *A*_*E*_ and *B*_*E*_, intraparticle diffusion model: *k*_*id*_ and *c*, and fractional power model: *k*_*fp*_ and *v*) under the different solution pH levels, corncob particle sizes, and *C*_*o*_ values assayed, together with the corresponding *r*^*2*^, *ASS*, *Sy*.*x*, and *AIC* values.

**Table 2:** Kinetic model parameters for DY27 biosorption onto corncob at different solution pH levels.

***Solution pH***	**1.5**	**2.0**	**2.5**	**3.0**	**4.0**	**5.0**	**6.0**
***q*_*e exp*_ [mg g^-1^]**	**35.1551 ± 0.8815**	**28.4783 ± 0.4683**	**20.5694 ± 0.4657**	**12.6606 ± 0.9735**	**6.900 ± 0.9896**	**4.2121 ± 0.79**	**1.5235 ± 0.08962**
***Pseudo-first-order***							
***q***_***e1***_	32.52 ± 1.0	25.65 ± 0.92	18.58 ± 0.66	11.51 ± 0.58	8.052 ± 0.409	4.716 ± 0.215	1.437 ± 0.059
***k***_***1***_	0.647 ± 0.0689	1.279 ± 0.204	1.222 ± 1.191	1.115 ± 0.8759	1.020 ± 0.2164	1.075 ± 0.2047	1.152 ± 0.1893
***r***^***2***^	0.9662	0.9238	0.9269	0.8724	0.8726	0.8950	0.9486
***ASS***	197.5	254.7	129.6	95.13	45.04	12.73	0.5502
***Sy*.*x***	2.008	2.280	1.626	1.393	0.9587	0.5098	0.1203
***AIC***	75.57	88.52	54.08	38.31	0.1721	-64.26	-164.8
***Pseudo-second-order***							
***q***_***e2***_	35.62 ± 0.87	28.02 ± 0.62	20.35 ± 0.46	12.68 ± 0.61	8.883 ± 0.394	5.195 ± 0.203	1.579 ± 0.061
***k***_***2***_	0.0259 ± 0.0031	0.06507 ± 0.0084	0.08452 ± 0.0109	0.1197 ± 0.03242	0.1577 ± 0.382	0.2840 ± 0.0616	0.9573 ± 0.1953
***r***^***2***^	0.9831	0.9794	0.9795	0.9162	0.9301	0.9443	0.9664
***ASS***	98.66	68.92	36.40	62.51	24.70	6.759	0.3595
***Sy*.*x***	1.419	1.186	0.8619	1.129	0.7100	0.3714	0.09727
***AIC***	40.16	21.86	-10.69	16.89	-30.46	-96.56	-181.8
***Elovich***							
***B***_***E***_	0.1921 ± 0.0226	0.2794 ± 0.0298	0.3802 ± 0.0403	0.5944 ± 0.089	0.8387 ± 0.1064	1.443 ± 1.3508	4.581 ± 0.771
***A***_***E***_	157.1 ± 74.14	448.1 ± 249.6	290 ± 157.1	140.9 ± 102.79	87.40 ± 52.73	54.80 ± 33.94	12.71 ± 9.506
***r***^***2***^	0.9181	0.9313	0.9316	0.8710	0.9035	0.9016	0.8806
***ASS***	479.0	229.8	121.2	96.22	34.12	11.94	1.279
***Sy*.*x***	3.126	2.165	1.573	1.401	0.8344	0.4936	0.1835
***AlC***	120.7	83.27	50.67	38.88	-13.99	-67.54	-131.0
***Intraparticle diffusion***							
***k***_***id***_	3.545 ± 0.887	2.523 ± 0.719	1.852 ± 0.52	1.18 ± 0.343	0.8446 ± 0.2268	0.4862 ± 0.1354	0.1504 ± 0.505
***c***	13.76 ± 2.87	13.97 ± 2.32	9.961 ± 1.68	5.951 ± 1.109	4.053 ± 0.733	2.415 ± 0.438	0.6664 ± 0.1654
***r***^***2***^	0.5688	0.5038	0.5117	0.4941	0.5340	0.5157	0.4887
***ASS***	2521	1659	865.7	377.3	164.7	58.76	5.478
***Sy*.*x***	7.173	5.819	4.203	2.775	1.833	1.095	0.3797
***AIC***	205.4	184.1	150.9	108.6	66.30	13.73	-72.86
***Fractional power***							
***k***_***fp***_	18.41 ± 1.53	17.56 ± 1.06	12.57 ± 0.77	7.588 ± 0.598	5.211 ± 0.372	3.086 ± 0.222	0.9003 ± 0.0932
***v***	0.1851 ± 0.0305	0.1465 ± 0.0234	0.1495 ± 0.0237	0.1563 ± 0.0301	0.1612 ± 0.0271	0.1580 ± 0.0276	0.1649 ± 0.0377
***r***^***2***^	0.8392	0.8669	0.8949	0.8665	0.8438	0.8371	0.7984
***ASS***	939.9	445.2	221.4	236.8	55.21	19.76	2.160
***Sy*.*x***	4.380	3.014	2.194	2.198	1.061	0.6350	0.2384
***AIC***	155.1	117.0	79.93	84.81	10.56	-41.85	-110.1

**Table 3:** Kinetic model parameters for DY27 biosorption onto corncob at different corncob particle sizes.

***Particle size* [mm]**	**0.297–0.500**	**0.500–0.800**	**0.800–1.000**	**1.000–1.180**	**1.18–1.410**	**1.410–1.700**	**1.700–2.000**
***q*_*e exp*_ [mg g^-1^]**	**35.1551 ± 0.8815**	**32.9090 ± 1.5308**	**33.5694 ± 1.4657**	**33.2058 ± 0.7393**	**31.2677 ± 0.9359**	**29.5313 ± 0.9634**	**26.7755 ± 0.04481**
***Pseudo-first-order***							
***q***_***e1***_	32.52 ± 1.0	31.27 ± 1.02	31.57 ± 0.95	30.58 ± 0.94	29.68 ± 0.95	28.05 ± 0.99	26.27 ± 0.89
***k***_***1***_	0.647 ± 0.0689	0.4052 ± 0.0386	0.2301 ± 0.0172	0.22124 ± 0.0249	0.1965 ± 0.0153	0.1519 ± 0.0133	0.1144 ± 0.0096
***r***^***2***^	0.9662	0.9724	0.9819	0.9810	0.9795	0.9765	0.9803
***ASS***	197.5	149.2	97.94	94.75	96.59	95.80	68.85
***Sy*.*x***	2.008	1.745	1.428	1.405	1.404	1.413	1.185
***AlC***	75.57	61.25	40.14	38.48	39.08	39.04	21.81
***Pseudo-second-order***							
***q***_***e2***_	35.62 ± 0.87	34.12 ± 0.77	34.78 ± 0.7	33.78 ± 0.66	32.93 ± 0.84	31.67 ± 0.98	30.52 ± 1.12
***k***_***2***_	0.0259 ± 0.0031	0.01665 ± 0.00162	0.008266 ± 0.0006	0.007761 ± 0.0006	0.007286 ± 0.0007	0.005537 ± 0.0006	0.004020 ± 0.0005
***r***^***2***^	0.9831	0.9891	0.9936	0.9941	0.9905	0.9879	0.9857
***ASS***	98.66	58.96	34.36	29.17	44.94	49.22	50.07
***Sy*.*x***	1.419	1.097	0.8460	0.7796	0.9577	1.013	1.011
***AIC***	40.16	13.91	-12.24	-20.42	0.06425	5.740	5.568
***Elovich***							
***B***_***E***_	0.1921 ± 0.0226	0.1806 ± 0.018	0.1508 ± 0.0105	0.1528 ± 0.0092	0.1541 ± 0.0103	0.1495 ± 0.0088	0.1425 ± 0.0098
***A***_***E***_	157.1 ± 74.14	63.58 ± 20.91	20.82 ± 3.6	17.87 ± 2.6	15.54 ± 2.43	9.606 ± 1.189	5.678 ± 0.712
***r***^***2***^	0.9181	0.9445	0.9774	0.9831	0.9794	0.9857	0.9828
***ASS***	479.0	300.5	122.1	84.09	96.86	58.22	60.20
***Sy*.*x***	3.126	2.476	1.595	1.324	1.406	1.101	1.108
***AlC***	120.7	96.97	51.17	32.52	39.22	14.13	14.97
***Intraparticle diffusion***							
***k***_***id***_	3.545 ± 0.887	3.696 ± 0.749	4.104 ± 0.551	3.993 ± 0.493	3.894 ± 0.466	3.74 ± 0.348	3.519 ± 0.263
***c***	13.76 ± 2.87	10.04 ± 2.421	5.642 ± 1.787	5.045 ± 1.598	4.619 ± 1.508	3.004 ± 1.13	1.640 ± 0.8494
***r***^***2***^	0.5688	0.6677	0.8240	0.8472	0.8521	0.9072	0.9367
***ASS***	2521	1799	951.7	760.5	696.8	378.6	221.2
***Sy*.*x***	7.173	6.059	4.453	3.980	3.771	2.808	2.125
***AIC***	205.4	188.2	153.8	142.6	139.9	107.7	81.34
***Fractional power***							
***k***_***fp***_	18.41 ± 1.53	14.66 ± 1.33	10.51 ± 1.007	9.733 ± 0.888	9.134 ± 0.851	7.239 ± 0.652	5.508 ± 0.532
***v***	0.1851 ± 0.0305	0.2243 ± 0.03143	0.2980 ± 0.0305	0.3074 ± 0.0286	0.3146 ± 0.0292	0.3526 ± 0.0272	0.3968 ± 0.0283
***r***^***2***^	0.8392	0.8678	0.9209	0.9330	0.9317	0.9535	0.9592
***ASS***	939.9	715.6	427.5	333.2	321.8	189.7	142.5
***Sy*.*x***	4.380	3.822	2.984	2.635	2.563	1.988	1.706
***AIC***	155.1	141.2	113.8	101.4	100.5	73.20	58.93

**Table 4:** Kinetic model parameters for DY27 biosorption onto corncob at different initial DY27 concentrations.

	***Initial concentration* [mg L^-1^]**								
	**7**	**13**	**28**	**50**	**74**	**85**	**100**	**145**	**293**
***q*_*e exp*_ [mg g^-1^]**	**6.519 ± 0.20**	**11.762 ± 0.25**	**19.897 ± 0.07**	**34.918 ± 0.88**	**48.425 ± 0.65**	**57.56 ± 0.268**	**67.14 ± 1.273**	**72.996 ± 1.40**	**73.04 ± 4.08**
***Pseudo-first-order***									
***q***_***e1***_	6.085 ± 0.216	10.43 ± 0.28	17.74 ± 0.88	31.51 ± 1.62	44.70 ± 1.92	54.48 ± 2.66	63.13 ± 3.32	69.35 ± 4.36	71.51 ± 3.43
***k***_***1***_	0.987 ± 0.142	0.825 ± 0.083	0.490 ± 0.076	0.510 ± 0.083	0.370 ± 0.045	0.365 ± 0.050	0.336 ± 0.052	0.351 ± 0.059	0.371 ± 0.049
***r***^***2***^	0.9362	0.9714	0.9238	0.9195	0.9538	0.9372	0.9373	0.9355	0.9552
***ASS***	12.29	18.08	127.3	435.8	501.0	957.1	1245	1291	929.3
***Sy*.*x***	0.5008	0.6074	1.612	2.982	3.198	4.420	5.319	5.829	5.012
***AIC***	-66.08	-46.39	53.18	115.9	123.0	156.0	158.3	145.6	130.4
***Pseudo-second-order***									
***q***_***e2***_	6.560 ± 0.191	11.46 ± 0.23	19.44 ± 0.6	34.58 ± 0.99	48.96 ± 1.05	58.90 ± 2.37	67.96 ± 2.58	74.87 ± 3.56	77.5 ± 3.33
***k***_***2***_	0.238 ± 0.039	0.101 ± 0.010	0.036 ± 0.005	0.021 ± 0.003	0.010 ± 0.001	0.009 ± 0.002	0.007 ± 0.001	0.007 ± 0.001	0.007 ± 0.001
***r***^***2***^	0.9683	0.9879	0.9763	0.9795	0.9903	0.9644	0.9723	0.9685	0.9711
***ASS***	6.109	7.666	39.71	111.0	105.2	541.7	549.0	630.7	600.4
***Sy*.*x***	0.3531	0.3955	0.9002	1.505	1.465	3.325	3.532	4.074	4.028
***AIC***	-101.7	-90.14	-6.254	46.17	43.45	127.0	120.6	117.0	113.3
***Elovich***									
***B***_***E***_	1.175 ± 0.165	0.604 ± 0.076	0.332 ± 0.023	0.186 ± 0.012	0.122 ± 0.008	0.106 ± 0.009	0.091 ± 0.007	0.0809 ± 0.007	0.081 ± 0.011
***A***_***E***_	82.35 ± 57.66	65.04 ± 34.79	53.84 ± 13.5	97.00 ± 22.43	75.57 ± 16.23	110.4 ± 33.78	116.4 ± 33.14	120.6 ± 34.1	148.5 ± 56.41
***r***^***2***^	0.8862	0.9064	0.9707	0.9754	0.9737	0.9510	0.9699	0.9650	0.9487
***ASS***	21.91	59.11	48.94	133.4	285.0	746.9	597.1	701.0	1064
***Sy*.*x***	0.6686	1.098	0.9994	1.650	2.412	3.904	3.684	4.295	5.362
***AlC***	-36.59	14.04	4.404	55.54	94.26	143.4	124.5	121.2	135.6
***Intraparticle diffusion***									
***k***_***id***_	0.5787 ± 0.18	1.120 ± 0.305	2.100 ± 0.397	3.757 ± 0.723	5.500 ± 0.943	6.402 ± 1.17	7.574 ± 1.328	8.793 ± 1.722	8.683 ± 1.915
***c***	3.166 ± 0.582	4.774 ± 0.988	6.434 ± 1.283	11.52 ± 2.335	13.25 ± 3.04	17.04 ± 3.79	18.3 ± 4.47	18.61 ± 5.17	21.85 ± 6.04
***r***^***2***^	0.4602	0.5258	0.6981	0.6902	0.7377	0.7120	0.7505	0.7379	0.6954
***ASS***	103.9	299.6	504.8	1677	2846	4387	4953	5250	6319
***Sy*.*x***	1.456	2.473	3.210	5.850	7.621	9.462	10.61	11.75	13.07
***AIC***	42.82	96.81	123.4	184.6	211.6	233.7	221.8	201.8	205.1
***Fractional power***									
***k***_***fp***_	3.980 ± 0.302	6.276 ± 0.536	9.080 ± 0.604	16.27 ± 1.1	19.99 ± 1.53	24.93 ± 1.98	27.97 ± 2.25	30.15 ± 2.65	33.14 ± 3.14
***v***	0.149 ± 0.029	0.175 ± 0.032	0.210 ± 0.024	0.210 ± 0.024	0.237 ± 0.026	0.227 ± 0.028	0.234 ± 0.026	0.2422 ± 0.030	0.2248 ± 0.033
***r***^***2***^	0.8111	0.8186	0.9116	0.9096	0.9104	0.8953	0.9188	0.9111	0.8935
***ASS***	36.37	114.6	147.7	489.0	972.0	1595	1612	1782	2209
***Sy*.*x***	0.8616	1.529	1.736	3.159	4.454	5.705	6.052	6.847	7.726
***AIC***	-10.73	47.80	60.75	121.8	156.8	182.1	170.2	158.5	164.1

The *r*^*2*^ values were higher with the pseudo-second-order kinetic model; and the corresponding *ASS*, *Sy*.*x*, and *AIC* values were lower than those obtained with the other four kinetic models. Therefore, the pseudo-second-order model is the most suitable for describing the experimental data obtained in this study, as shown by the solid lines in Figs 4–6. In addition, the equilibrium biosorption capacity calculated from this model (*q*_*e2*_) matched the experimental value (*q*_*e exp*_) closely.

The pseudo-second-order model has also been successfully used to describe the biosorption kinetics of other dye-biosorbent systems. Examples include red 27 onto the leaves of *Eichhornia crassipes* [16]; direct red-31 and direct orange-26 onto rice husks [18]; methylene blue onto tamarind fruit shell [39]; crystal violet onto *Artocarpus altilis* (breadfruit) skin [30]; acid orange 7, basic red 46, and basic blue 3 onto *Spirogyra* sp. [43]; and reactive black 5 onto *Laminaria* sp. [44].

Tables 2–4 show the variation in the pseudo-second-order model rate constant (*k*_*2*_) according to the solution pH, corncob particle size, and *C*_*o*_. A lower *k*_*2*_ value means a longer time needed to reach the equilibrium biosorption capacity under the given experimental condition, because *k*_*2*_ plays the key role of a time-scaling factor in the corresponding kinetic equation [45]. The same trend was observed in other dye biosorption systems [16, 18, 41, 44].

### DY27 biosorption isotherms and their mathematical modeling

**Fig 7:**
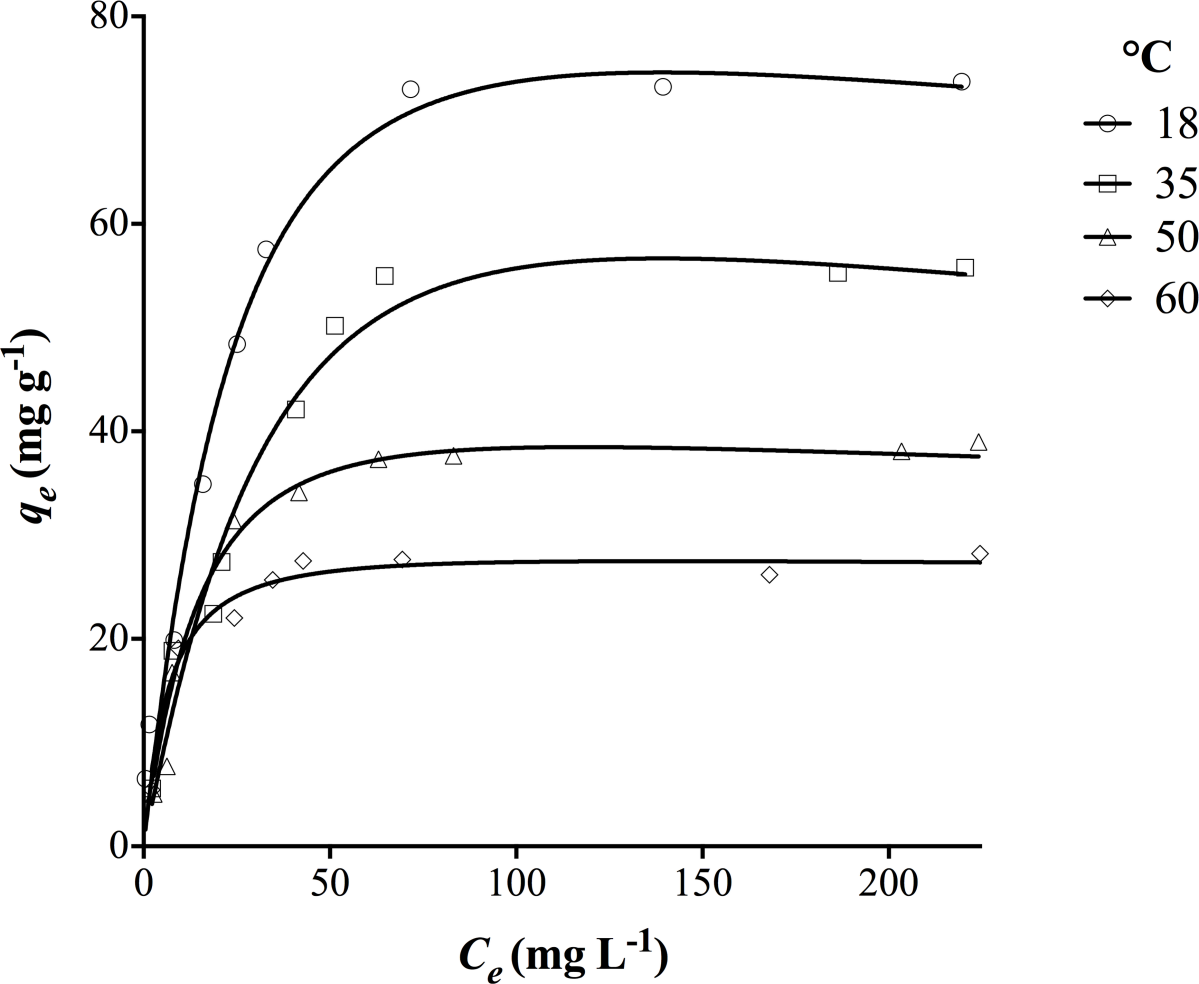
Isotherms of DY27 biosorption by corncob at different temperatures.

Fig 7 illustrates the biosorption isotherms of DY27 by corncob at pH = 1.5 and different temperatures (18, 35, 50, and 60°C). The obtained isotherms are regular, smooth, and concave to the x-axis (concentration axis). The concave shape and initial slope indicate that as more biosorption binding sites are occupied, it becomes more difficult for the remaining dye molecules to find available active sites on the corncob surface, which suggests a progressive saturation of corncob with DY27 [16, 46].

The DY27 biosorption performance decreased with increasing temperature from 18 to 60°C, revealing the exothermic nature of this process. This lower biosorption at higher temperature could be attributed to many possible factors: increased solubility of DY27 in water, stronger dye-solvent interaction than that between DY27 molecules and corncob, increased Brownian movement of DY27 molecules in solution, the breaking of existing intermolecular hydrogen bonding between DY27 and corncob, and/or damaged active biosorption sites that decrease the surface activity [1, 47]. A decrease in the dye biosorption performance at higher temperatures has also been reported for the biosorption of FD&C red No. 40 and acid blue 9 by *Spirulina platensis* [48]; acid blue 9, food yellow 3, and FD&C yellow No. 5 by chitosan [49]; methylene blue, eriochrome black T, and alizarin S by prickly pear cactus (*Opuntia ficus indica*) cladodes [50]; and astrazon red by the leaves of *Posidonia oceanica* (Linnaeus) Delile [51], among others.

The DY27 biosorption isotherms were modeled with several two-parameter (Langmuir, Freundlich, Temkin, Halsey, and Dubinin-Radushkevich) and three-parameter (Sips, Radke-Prausnitz, Redlich-Peterson, and Toth) models. The parameters obtained from nonlinear regression analysis for different temperatures, along with their corresponding *r*^*2*^, *ASS*, *Sy*.*x*, and *AIC* values are shown in Tables 5–8. The equilibrium biosorption of DY27 onto corncob is best described by the Redlich-Peterson model, as it yielded a higher *r*^*2*^ value and lower *ASS*, *Sy*.*x* and *AIC* values at each assayed temperature compared to the other isotherm models. The good agreement between this model and experimental results are shown in Fig 7 (lines and points, respectively).

**Table 5:** Parameters of the isotherm models for the biosorption at 18°C [*q*_*m exp*_: 73.71 ± 4.8 mg g^-1^].

Two-parameter isotherms Base 100		Three-parameter isotherms	
**Langmuir**		**Sips**	
**K_*L*_ [L mg^-1^]**	0.05430 ± 0.01092	**K**_***S***_ **[(mg L**^**-1**^**)**^**-1/ ns**^**]**	0.05871 ± 0.01076
**q_*m*_ [mg g^-1^]**	81.75 ± 4.74	**q**_***m***_ **[mg g**^**-1**^**]**	79.61 ± 5.87
**r^2^**	0.9728	**n**_***S***_	0.8127 ± 0.1872
***ASS***	295.5	**r**^**2**^	0.9748
**S*y*.*x***	4.252	***ASS***	450.2
***AIC***	85.60	**S*y*.*x***	4.331
**Freundlich**		***AIC***	85.79
**K_*F*_ [(mg g^-1^) (mg L^-1^)^-1/nF^]**	16.00 ± 4.21	**Radke-Prausnitz**	
**n_*F*_**	3.267 ± 0.619	**A**_***R***_ **[L g**^**-1**^**]**	5.029 ± 2.35
**r^2^**	0.8922	**R**_***R***_ **[L mg**^**-1**^**]**	83.16 ± 5.32
***ASS***	1913	**B**_***R***_	0.0029 ± 0.1801
**S*y*.*x***	8.748	**r**^**2**^	0.9700
***AIC***	122.1	***ASS***	7691
**Temkin**		**S*y*.*x***	2.777
**A_*T*_ [L mg^-1^]**	1.071 ± 0.1061	***AIC***	2048
**B_*T*_ [J mol^-1^]**	72.45 ± 1.56	**Redlich-Peterson**	
**r^2^**	0.8919	**K**_***RP***_ **[L g**^**-1**^**]**	3.245 ± 0.033
***ASS***	26223	**A**_***RP***_ **[(L mg**^**-1**^**)**^**BRP**^**]**	0.01415 ± 0.0001
**S*y*.*x***	5.126	**B**_***RP***_	0.991 ± 0.009
***AIC***	3273	**r**^**2**^	0.9965
**Halsey**		***ASS***	282.7
**K_*H*_ [(L g^-1^)^-1/nH^]**	1.218E-006 ± 0.888E-006	**S*y*.*x***	0.9672
**n_*H*_**	-0.2318 ± 0.0091	***AIC***	-61.61
**r^2^**	0.7992	**Toth**	
***ASS***	47971	**q**_***m***_ **[mg g**^**-1**^**]**	75.03 ± 3.47
**S*y*.*x***	6.933	**b**_***T***_ **[(L mg**^**-1**^ **)**^**-1/ nT**^**]**	0.03345 ± 0.00559
***AIC***	3877	**n**_***T***_	0.4529 ± 0.1801
**Dubinin-Radushkevich**		**r**^**2**^	0.9826
**q_*m*_ [mg g^-1^]**	73.73 ± 0.23	***ASS***	302.9
**B_*DR*_ [mol^2^ J^-2^]**	0.0001679 ± 0.0015159	**S*y*.*x***	4.552
**r^2^**	0.9573	***AIC***	75.09
***ASS***	9281		
**S*y*.*x***	4.350		
***AIC***	2234		

**Table 6:** Parameters of the isotherm models for the biosorption at 35°C [*q*_*m exp*_: 55.79 ± 4.29 mg g^-1^].

Two-parameter isotherms Base 100		Three-parameter isotherms	
**Langmuir**		**Sips**	
**K_*L*_ [L mg^-1^]**	0.04341 ± 0.0379	**K**_***S***_ **[(mg L**^**-1**^**)**^**-1/ ns**^**]**	0.04834 ± 0.01061
**q_*m*_ [mg g^-1^]**	65.03 ± 5.08	**q**_***m***_ **[mg g**^**-1**^**]**	60.65 ± 5.67
**r^2^**	0.9425	**n**_***S***_	0.7726 ± 0.2039
***ASS***	419.6	**r**^**2**^	0.9501
**S*y*.*x***	4.025	***ASS***	424.7
***AIC***	85.28	**S*y*.*x***	4.207
**Freundlich**		***AIC***	84.22
**K_*F*_ [(mg g^-1^) (mg L^-1^)^-1/nF^]**	11.63 ± 3.822	**Radke-Prausnitz**	
**n_*F*_**	3.210 ± 0.745	**A**_***R***_ **[L g**^**-1**^**]**	3.400 ± 2.35
**r^2^**	0.822	**R**_***R***_ **[L mg**^**-1**^**]**	15.38 ± 0.76
***ASS***	1516	**B**_***R***_	0.02599 ± 0.0103
**S*y*.*x***	7.787	**r**^**2**^	0.7939
***AIC***	115.8	***ASS***	29491
**Temkin**		**S*y*.*x***	5.439
**A_*T*_ [L mg^-1^]**	0.5905 ± 0.0522	***AIC***	3392
**B_*T*_ [J mol^-1^]**	84.60 ± 1.86	**Redlich-Peterson**	
**r^2^**	0.8879	**K**_***RP***_ **[L g**^**-1**^**]**	1.816 ± 0.02
***ASS***	16040	**A**_***RP***_ **[(L mg**^**-1**^**)**^**BRP**^**]**	0.005964 ± 0.0001
**S*y*.*x***	4.009	**B**_***RP***_	0.999 ± 0.001
***AIC***	2781	**r**^**2**^	0.9947
**Halsey**		***ASS***	358.8
**K_*H*_ [(L g^-1^)^-1/nH^]**	2.710E-005 ± 1.637E-005	**S*y*.*x***	0.8724
**n_*H*_**	-0.2599 ± 0.0123	***AIC***	-268.0
**r^2^**	0.7939	**Toth**	
***ASS***	29491	**q**_***m***_ **[mg g**^**-1**^**]**	56.6 ± 3.25
**S*y*.*x***	5.436	**b**_***T***_ **[(L mg**^**-1**^ **)**^**-1/ nT**^**]**	0.0252 ± 0.00393
***AIC***	3390	**n**_***T***_	0.3625 ± 0.1617
**Dubinin-Radushkevich**		**r**^**2**^	0.9646
**q_*m*_ [mg g^-1^]**	56.27 ± 0.23	***ASS***	431.0
**B_*DR*_ [mol^2^ J^-2^]**	0.0002555 ± 0.0015159	**S*y*.*x***	4.541
**r^2^**	0.9624	***AIC***	74.92
***ASS***	5382		
**S*y*.*x***	4.322		
***AIC***	1689		

**Table 7:** Parameters of the isotherm models for the biosorption at 50°C [q_m exp_: 38.99 ± 3.9 mg g^-1^].

Two-parameter isotherms Base 100		Three-parameter isotherms	
**Langmuir**		**Sips**	
**K_*L*_ [L mg^-1^]**	0.07311 ± 0.0379	**K**_***S***_ **[(mg L**^**-1**^**)**^**-1/ ns**^**]**	0.09408 ± 0.011
**q_*m*_ [mg g^-1^]**	43.19 ± 1.01808	**q**_***m***_ **[mg g**^**-1**^**]**	38.73 ± 1.35
**r^2^**	0.9524	**n**_***S***_	0.5929 ± 0.0961
***ASS***	216.4	**r**^**2**^	0.9834
**S*y*.*x***	2.042	***ASS***	275.44
***AIC***	63.23	**S*y*.*x***	2.773
**Freundlich**		***AIC***	37.56
**K_*F*_ [(mg g^-1^) (mg L^-1^)^-1/nF^]**	10.09 ± 3.476	**Radke-Prausnitz**	
**n_*F*_**	3.681 ± 1.022	**A**_***R***_ **[L g**^**-1**^**]**	4.308 ± 0.115
**r^2^**	0.7713	**R**_***R***_ **[L mg**^**-1**^**]**	41.12 ± 0.15
***ASS***	1039	**B**_***R***_	1.541E-016 ± 0.0103
**S*y*.*x***	6.445	**r**^**2**^	0.9422
***AIC***	105.6	***ASS***	1886
**Temkin**		**S*y*.*x***	2.375
**A_*T*_ [L mg^-1^]**	6.114 ± 1.351	***AIC***	642.3
**B_*T*_ [J mol^-1^]**	188.6 ± 6.6	**Redlich-Peterson**	
**r^2^**	0.76	**K**_***RP***_ **[L g**^**-1**^**]**	2.685 ± 0.037
***ASS***	7827	**A**_***RP***_ **[(L mg**^**-1**^**)**^**BRP**^**]**	0.03161 ± 0.00114
**S*y*.*x***	2.800	**B**_***RP***_	0.995 ± 0.004
***AIC***	2064	**r**^**2**^	0.9901
**Halsey**		***ASS***	214.1
**K_*H*_ [(L g^-1^)^-1/nH^]**	8.976E-009 ± 1.266E-009	**S*y*.*x***	0.5702
**n_*H*_**	-0.1553 ± 0.0123	***AIC***	-1119
**r^2^**	0.6827	**Toth**	
***ASS***	10347	**q**_***m***_ **[mg g**^**-1**^**]**	38.32 ± 1.39
**S*y*.*x***	3.220	**b**_***T***_ **[(L mg**^**-1**^ **)**^**-1/ nT**^**]**	0.04885 ± 0.00709
***AIC***	2343	**n**_***T***_	0.4321 ± 0.1421
**Dubinin-Radushkevich**		**r**^**2**^	0.9817
**q_*m*_ [mg g^-1^]**	38.04 ± 0.09	***ASS***	283.07
**B_*DR*_ [mol^2^ J^-2^]**	8.049E005± 0.195E005	**S*y*.*x***	2.460
**r^2^**	0.9547	***AIC***	40.16
***ASS***	1478		
**S*y*.*x***	2.217		
***AIC***	397.0		

**Table 8:** Parameters of the isotherm models for the biosorption at 60°C [q_m exp_: 28.23 ± 1.0 mg g^-1^].

Two-parameter isotherms Base 100		Three-parameter isotherms	
**Langmuir**		**Sips**	
**K_*L*_ [L mg^-1^]**	0.1615 ± 0.0409	**K**_***S***_ **[(mg L**^**-1**^**)**^**-1/ ns**^**]**	0.1711 ± 0.0304
**q_*m*_ [mg g^-1^]**	29.30 ± 1.28	**q**_***m***_ **[mg g**^**-1**^**]**	27.80 ± 1.15
**r^2^**	0.9490	**n**_***S***_	0.7354 ± 0.1472
***ASS***	42.63	**r**^**2**^	0.9657
**S*y*.*x***	1.425	***ASS***	63.33
***AIC***	30.49	**S*y*.*x***	1.697
**Freundlich**		***AIC***	23.89
**K_*F*_ [(mg g^-1^) (mg L^-1^)^-1/nF^]**	11.70 ± 3.213	**Radke-Prausnitz**	
**n_*F*_**	5.543 ± 1.956	**A**_***R***_ **[L g**^**-1**^**]**	5.554 ± 0.158
**r^2^**	0.6886	**R**_***R***_ **[L mg**^**-1**^**]**	28.6 ± 0.49
***ASS***	386.8	**B**_***R***_	1.717E-016 ±000.0103
**S*y*.*x***	4.193	***r***^***2***^	0.9757
***AIC***	73.92	***ASS***	212.6
**Temkin**		**S*y*.*x***	0.4617
**A_*T*_ [L mg^-1^]**	116.6 ± 39.67	***AIC***	-1540
**B_*T*_ [J mol^-1^]**	377.3 ± 13.8	**Redlich-Peterson**	
**r^2^**	0.7404	**K**_***RP***_ **[L g**^**-1**^**]**	4.158 ± 0.037
***ASS***	2266	**A**_***RP***_ **[(L mg**^**-1**^**)**^**BRP**^**]**	0.113 ± 0.0017
**S*y*.*x***	1.507	**B**_***RP***_	0.989 ± 0.001
***AIC***	824.0	**r**^**2**^	0.9961
**Halsey**		***ASS***	34.04
**K_*H*_ [(L g^-1^)^-1/nH^]**	4.236E-012 ± 2.32E-012	**S*y*.*x***	0.1848
**n_*H*_**	-0.1066 ± 0.0051	***AIC***	-3372
**r^2^**	0.6833	**Toth**	
***ASS***	2765	**q**_***m***_ **[mg g**^**-1**^**]**	27.71 ± 1.29
**S*y*.*x***	1.664	**b**_***T***_ **[(L mg**^**-1**^ **)**^**-1/ nT**^**]**	0.11 ± 0.03423
***AIC***	1023	**n**_***T***_	0.6536 ± 0.2326
**Dubinin-Radushkevich**		**r**^**2**^	0.9636
**q_*m*_ [mg g^-1^]**	27.15 ± 0.06	***ASS***	45.19
**B_*DR*_ [mol^2^ J^-2^]**	2.789E005± 0.095E005	**S*y*.*x***	1.467
**r^2^**	0.9052	***AIC***	25.29
***ASS***	827.4		
**S*y*.*x***	2.9105		
***AIC***	-183.5		

The Redlich-Peterson isotherm model is a hybrid model of Langmuir and Freundlich models, and it incorporates three parameters into a single empirical equation. This versatile model has been successfully used to represent biosorption equilibria over a wide concentration range, and can be applied to both homogeneous and heterogeneous systems [52]. Examples include the biosorption of FD&C red No. 40 onto chitosan [53], acid red 18 and FD&C blue No. 2 onto chitosan films [54], reactive blue 19 onto cross-linked chitosan/oil palm ash composite beads [55], Gemazol turquoise blue-G reactive dye onto dried sugar beet pulp [56], and methylene blue onto activated carbons [57].

The maximum experimental biosorption capacity (*q*_*m exp*_) was found to be 73.7 mg g^-1^ at 18°C (Table 5). As mentioned above, to the best of our knowledge, this is the first report of using a biosorbent to remove DY27 from aqueous solutions. The only previous work on DY27 removal used an inorganic adsorbent, namely poorly crystalline hydroxyapatite prepared via a precipitation method [26]. In that study, the maximum adsorption occurred at pH 3.5 to be 6.8 mg g^-1^, while the corresponding value predicted by the Langmuir model was 89.26 mg g^-1^ [26].

The above results indicate that corncob may be used as a low-cost, abundant, and green biosorbent with high capacity to remove DY27 from contaminated water and wastewater.

### Thermodynamics of DY27 biosorption

The DY27 biosorption isotherm data at different temperatures were used to calculate important thermodynamic parameters, namely changes in Gibbs free energy (*ΔG*°), enthalpy (*ΔH*°), and entropy (*ΔS*°), in order to provide in-depth information about the inherent energetic changes during the biosorption process [37, 58]. According to Table 9, the *ΔG*° values were negative at all the temperatures (18–60°C), indicating that the biosorption of DY27 onto corncob was spontaneous and thermodynamically favorable (i.e., there is an affinity of corncob towards the DY27 dye) [37, 44]. These *ΔG*° values are within the general range of -20 to 0 kJ mol^-1^ for physisorption processes [37, 55].

**Table 9:** Thermodynamic parameters for the biosorption.

T [°C]	Δ*G*° [J mol^-1^]	Δ*S*° [J mol^-1^ K^-1^]	Δ*H*° [J mol^-1^]
**18**	-2135.00 ± 39.03	25.956 ± 0.6311	-9692.09 ± 39.03
**35**	-1522.24 ± 34.97		-9520.58 ± 34.97
**50**	-1136.06 ± 29.24		-9523.74 ± 29.24
**60**	-1074.53 ± 26.87		-9721.77 ± 26.95

The *ΔG*° value was more negative at 18°C, indicating that the biosorption was more energetically favorable at lower temperatures [1, 37]. This is due to the positive *ΔS*° value that indicates increased randomness at the solid-liquid interface after biosorption [37]. The positive *ΔS*° value also suggests the possibility of some structural changes or readjustments in the DY27-corncob complex [1].

The negative *ΔH*° values indicate that the biosorption is an exothermic process, in agreement with the observed decrease in biosorption as the temperature increases. The magnitude of *ΔH*° value confirms that it is a physical sorption process (physisorption), for which the values of |*ΔH*°| are usually lower than 84 kJ mol^-1^ [37]. It is well known that the physisorption is exothermic and enhanced at lower temperature, because the interaction between adsorbates and active sites is weakened at higher temperatures [59].

### FTIR analysis

**Fig 8:**
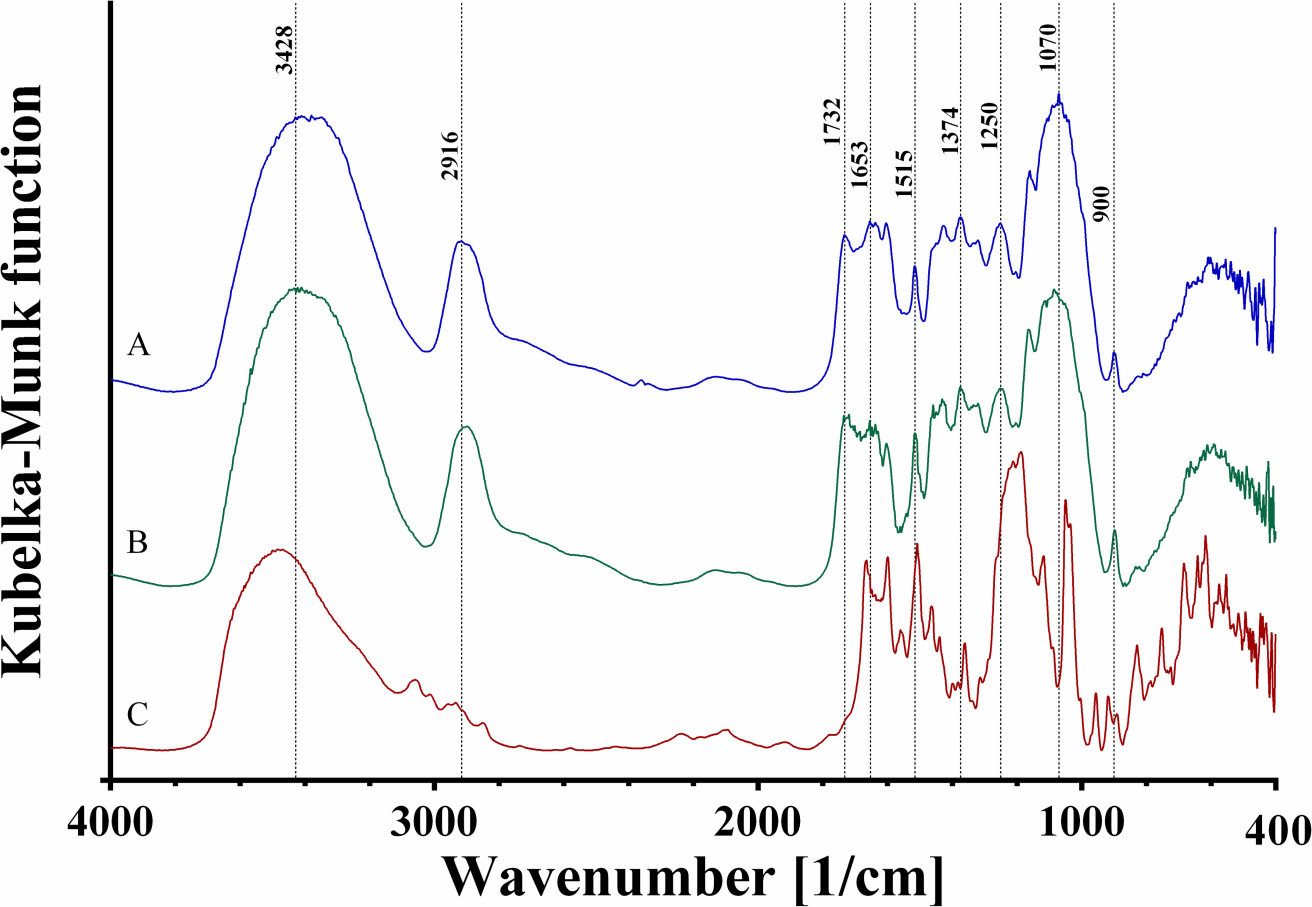
FTIR spectra of (A) native corncob, (B) DY27-loaded corncob, and (C) DY27 dye.

FTIR spectra were collected for native and DY27-loaded corncob samples, in order to examine the interactions between dye molecules with the functional groups on the corncob. Fig 8 displays the spectra for the corncob samples and that of DY27 alone, and Table 10 lists the peak positions of the major absorption bands.

**Table 10:** Summary of infrared spectral bands observed in the native corncob, DY27-loaded corncob, and DY27 dye.

IR band frequency [cm^-1^]			Functional group assignment	Reference
Native corncob	DY27-loaded corncob	DY27 dye		
3428	3421	3498	O-H stretching	[60]
2916	2910	-	Symmetric stretching of C-H	[61]
1732	1734	-	C = O stretching vibration of hemicellulose	[62]
1653	1654	1664	C = O stretching of amide I of proteins	[63]
1603	1603	1598	Aromatic skeletal vibration + C = O stretching	[64]
1515	1514	-	Aromatic skeletal vibration of lignin	[64]
-	-	1507	N = N stretching	[65]
-	1458	1464	C = C stretching of aromatic rings of dye	[65]
1426	1430	1438	C-H in plane deformation with aromatic ring stretching	[66]
1374	1375	-	Symmetric C-H deformation of cellulose	[67]
1250	1248	-	C-O stretching of hemicellulose	[68]
-	-	1211, 1188	Amine C-N stretching	[69]
1160	1163	-	C-O-C stretching of glycosidic linkages	[70]
1070	1085	-	C-O deformation of secondary alcohols and aliphatic ethers	[64]
1049 shoulder	1051 shoulder	1049	C-O stretching vibrations of cellulose S = O stretching of sulfonate groups	[70] [63]
900	898	-	Antisymmetric out-of-plane ring stretching of cellulose	[66]

Cellulose, hemicellulose, and lignin are the main constituents of corncob. They contain functional groups that exhibit common absorption bands, including the OH stretching at 3428 cm^-1^ [60] and the symmetric stretching of aliphatic chains (-CH) at 2916 cm^-1^ [61]. Absorption bands related mainly to cellulose were observed at 1374, 1160, and 900 cm^-1^, which are associated with the symmetric C-H deformations, C-O-C stretching of glycosidic bonds, and antisymmetric out-of-plane ring stretching, respectively [66, 67, 70]. Two characteristic absorption bands of hemicellulose were identified, the first one at 1732 cm^-1^ is associated with the stretching vibration of C = O group [62], and the second at 1250 cm^-1^ is attributed to the stretching of C-O bond [68, 71]. Representative absorption bands of lignin were found at 1515, 1603, 1426, and 1070 cm^-1^, which correspond to the aromatic skeletal vibration, the aromatic skeletal vibration breathing with C = O stretching [64, 66, 72], the C-H in-plane deformation with aromatic ring stretching, and the C-O deformations of secondary alcohols and aliphatic ethers [64], respectively. The characteristic absorption band of amide I C = O stretching at 1653 cm^-1^ [63] was also detected in the spectrum of native biosorbent, and this band most probably corresponds to the amide groups in the proteins.

After loading DY27 onto the native biosorbent, the FTIR spectrum showed a few changes. The relative intensity decreased for the bands at 1653, 1603, 1374, and 1070 cm^-1^. Based on the assignments described above, these changes indicate the involvement of the proteins, cellulose, and lignin of corncob in the biosorption of DY27 dye molecules. Additionally, the absorption band at 1514 cm^-1^ became more intense, which can be explained by the combination between the band corresponding to the aromatic skeletal vibration of lignin at 1514 cm^-1^ and a characteristic absorption band of DY27 (the stretching of the azo functional group) at 1507 cm^-1^ [65]. A new absorption band appeared at 1458 cm^-1^, which could be related again to the presence of the DY27 dye, because this wavenumber is close to the C = C stretching frequency of its aromatic ring at 1464 cm^-1^ [65]. The presence of these functional groups belonging to DY27 in the dye-loaded corncob may come from the dye molecules that either do not participate directly in the biosorption process or were biosorbed through weak forces.

Dyes with linear molecular structures (including DY27) are used mainly for dyeing paper. The paper dyeing process basically involves the penetration of dye molecules into the capillary spaces of cellulose, followed by dye adsorption on the fiber surface. The adsorption is driven by the effects of charge, precipitation, and intermolecular forces. It is known that lignin has a considerable influence on the dyeability, but not the hemicellulose [73]. This finding in paper dyeing is consistent with the analysis of the FTIR spectra of DY27 biosorbed on corncob.

### CSLM analysis

CSLM is a powerful optical technique for visualizing the structure of biopolymer mixtures. In CSLM, optical contrast is obtained by differences in fluorescence, either by auto-fluorescence of the material or by the addition of specific fluorescent dyes (fluorochromes) [74]. Each fluorochrome has its own particular emission wavelength [75]. In the present work, two fluorochromes were used: fluorescein and calcofluor white M2R.

The auto-fluorescence of plant cells is mainly due to the presence of lignin and chlorophylls [76]. In the present work, the maximum emission wavelength (λ_em max_) for lignin auto-fluorescence was observed at 501 nm (data not shown), which agrees with that reported by Rosas-Hernández (500 nm) [77]. Likewise, the peak of maximum emission for DY27 was found at 598 nm (data not shown).

The proteins were examined by staining DY27-loaded corncob samples with fluorescein, which binds covalently to the primary amine groups on the proteins. The λ_em max_ for fluorescein was found at 519 nm, which is close to those reported by van de Velde et al. (518 nm) [74], Becker et al. (530 nm) [78], and the datasheet from the manufacturer (514 nm) [79].

**Fig 9:**
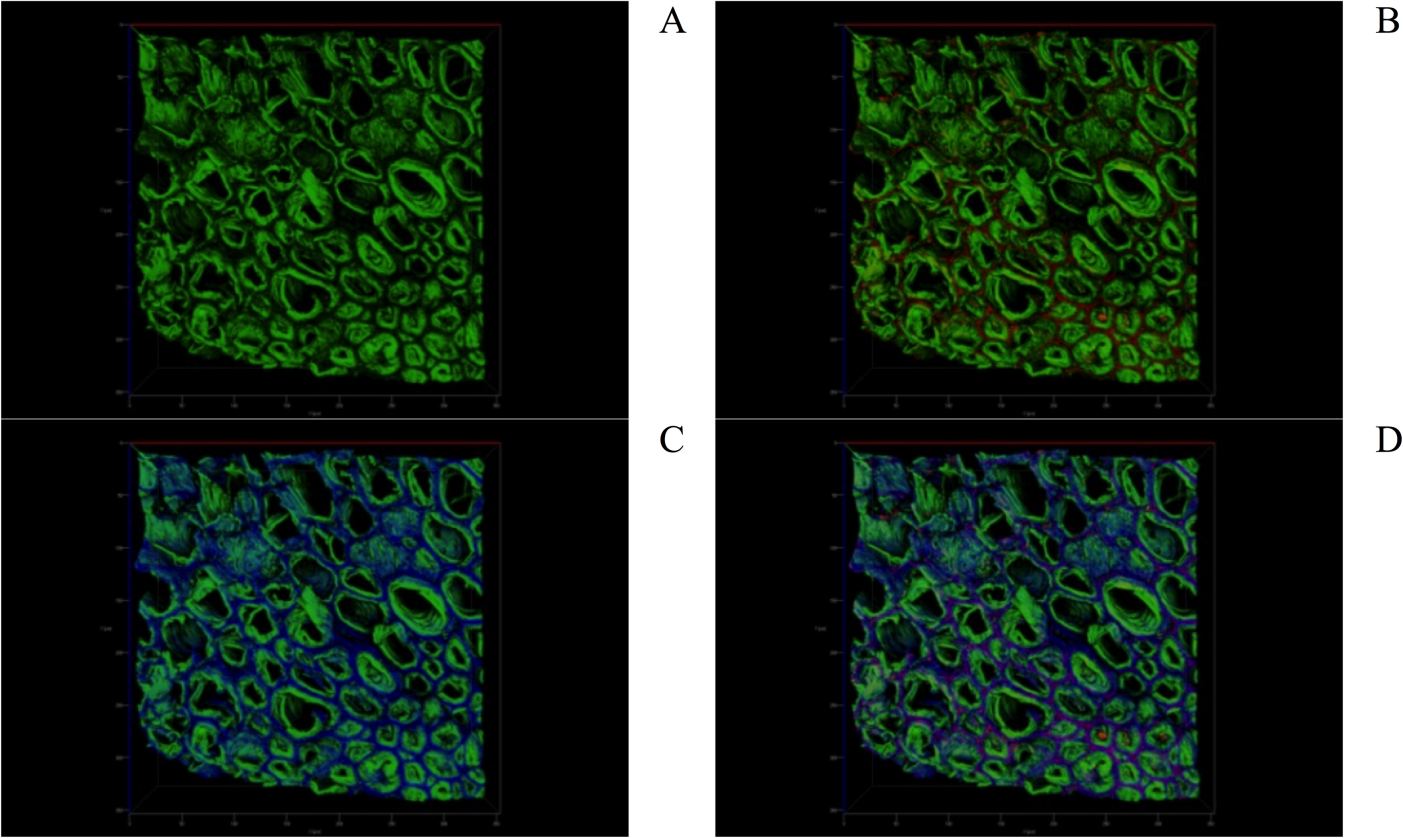
CSLM images of a) lignin (green); b) lignin and DY27 dye in green and red colors, respectively; and c) fluorescein-induced fluorescence of corncob proteins in blue color. d) An overlay image showing the interaction between the DY27 dye and the corncob proteins in violet color.

Fig 9 shows CSLM images of DY27-loaded corncob stained with fluorescein. The auto-fluorescence of lignin (green color, Fig 9A), the fluorescence of DY27 dye on the corncob surface (red color, Fig 9B), and the fluorescein-induced fluorescence of corncob proteins (blue color, Fig 9C) can be observed. Furthermore, the interaction between DY27 with the corncob proteins was evidenced by a violet color in the overlay image (Fig 9D) produced by the combination of the red and blue colors from the DY27 dye and the fluorescein-induced fluorescence of proteins, respectively. These results clearly demonstrate that the DY27 dye binds to the proteins present in corncob.

The cellulose was examined by staining DY27-loaded corncob samples with calcofluor white M2R solution. This fluorochrome emits fluorescence when activated by ultraviolet radiation and has an affinity for the cellulose and chitin in cell walls [80, 81]. The λ_em max_ for calcofluor white was observed at 463 nm (data not shown), which is close to that reported in the datasheet from the manufacturer (433 nm) [82].

**Fig 10:**
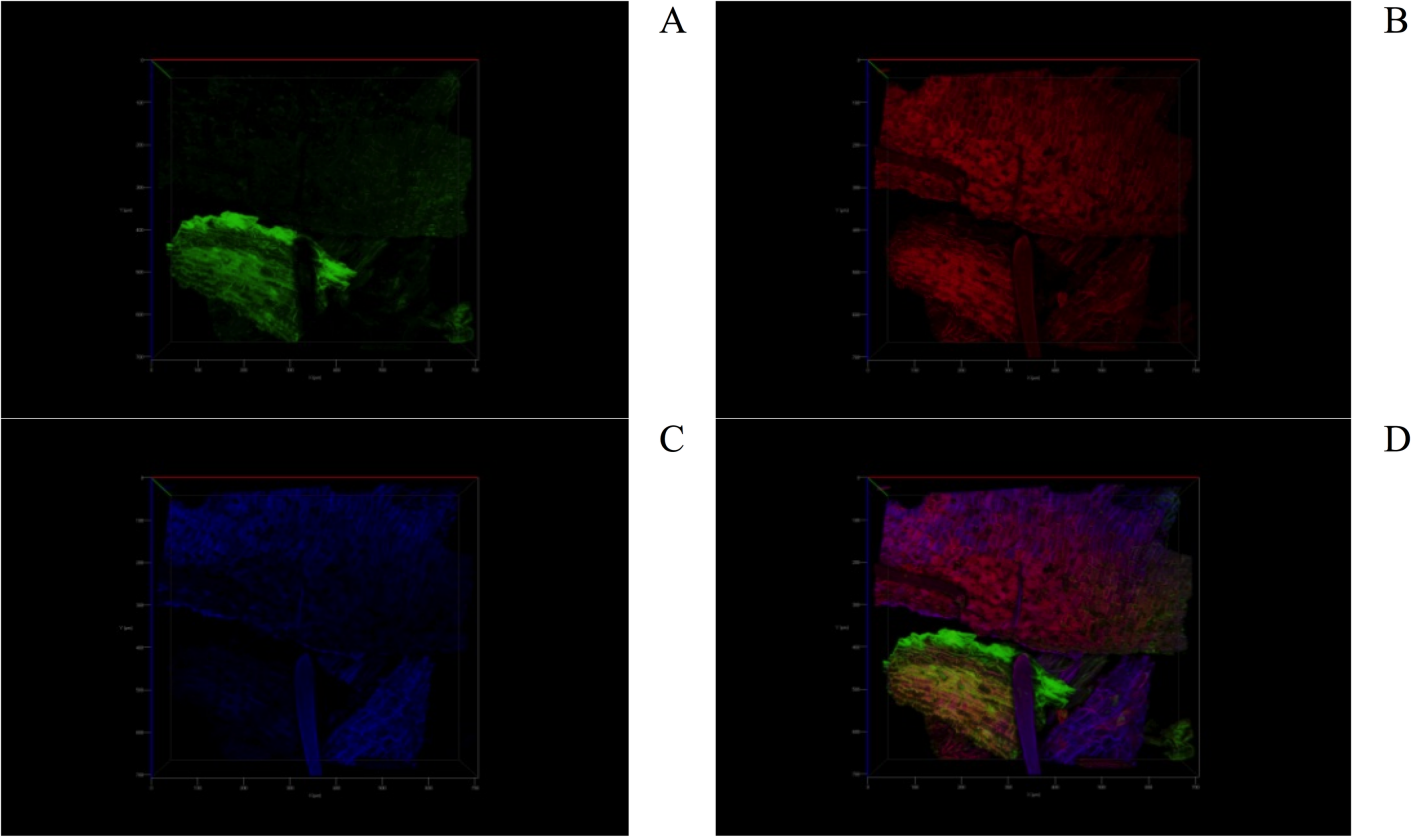
CSLM images of a) lignin (green); b) DY27 dye on the corncob surface in red color; and c) calcofluor white-induced fluorescence of corncob cellulose in blue color. d) An overlay image showing the interaction between the DY27 dye and the corncob cellulose in violet color.

Fig 10 shows CSLM images of DY27-loaded corncob stained with calcofluor white. The auto-fluorescence of lignin is shown in green color (Fig 10A), the fluorescence of DY27 on the corncob surface in red (Fig 10B), and the calcofluor-induced fluorescence of corncob cellulose in blue (Fig 10C). The binding of DY27 onto the corncob cellulose was revealed by a violet color in the overlay image (Fig 10D), which is also due to the combination of the red and blue colors from DY27 and the calcofluor-induced fluorescence of cellulose, respectively. These results indicate that the DY27 dye also binds to the corncob cellulose.

The above findings clearly show that proteins and lignocellulose in the corncob play a crucial role in DY27 biosorption from aqueous solutions, which is consistent with the FTIR findings in this study.

### DY27 desorption studies

**Fig 11:**
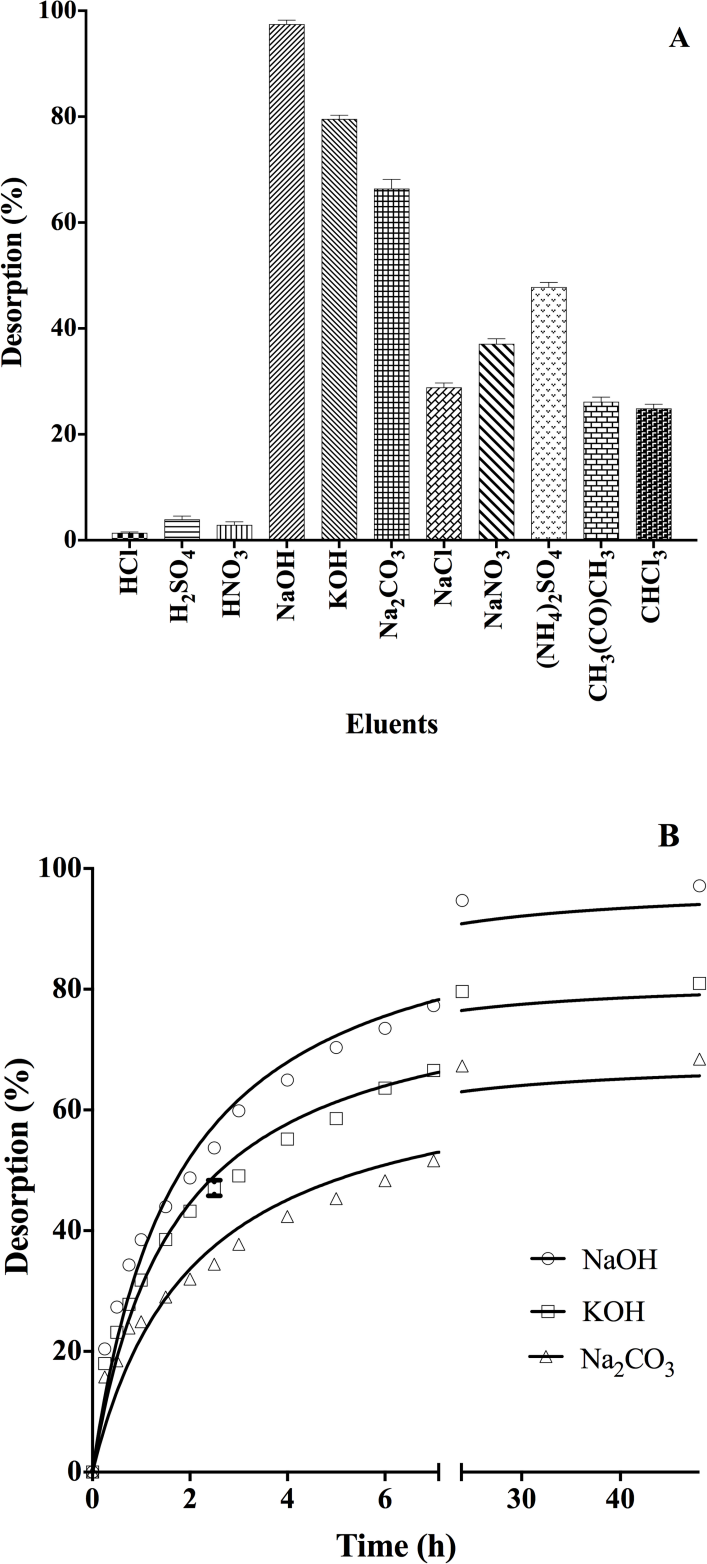
Effect of eluents on (A) DY27 desorption and (B) desorption kinetics.

Desorption of the dye from the DY27-loaded corncob was tested using several chemical agents, including acid, alkali, neutral salt, and organic solvent solutions. The performances of these eluent solutions are shown in Fig 11A. The highest DY27 desorption percentage was observed for strong alkali solutions, followed by weak alkaline, neutral salt, weak acid, organic solvent, and lastly strong acid solutions. Therefore, the DY27 desorption percentage depends strongly on the eluent pH: the higher the pH, the higher the desorption percentage. This trend may be explained on the basis of electrostatic repulsion between the negatively charged sites on the corncob biomass and the anionic DY27 molecules [16, 83]. The strong acid solutions performed poorly in the desorption, eluting only negligible amounts (< 4%) of biosorbed DY27. Therefore, we selected the alkaline solutions (NaOH, KOH, and Na_2_CO_3_) for further kinetic studies of the desorption process.

Fig 11B displays the DY27 desorption percentage as a function of desorption time. It is evident that for all three alkaline solutions, the desorption was initially very fast and became slower towards equilibrium. The highest rate and extent of DY27 desorption were achieved with 0.1 M NaOH solution, with a desorption efficiency of approximately 98%. These results clearly indicate that 0.1 M NaOH is the best eluent for DY27 desorption from the corncob-based biosorbent.

## Conclusions

The present work clearly demonstrates the feasibility of utilizing corncob as a novel, effective, efficient, eco-friendly, and inexpensive biosorbent for the removal of DY27 dye from aqueous solutions. The DY27 biosorption performance of corncob was affected significantly by solution pH, corncob particle size, shaking contact time, and initial dye concentration. The kinetic data of biosorption were accurately described by the pseudo-second-order model, and the biosorption isotherms were well predicted by the Redlich-Peterson model. Thermodynamic study indicated that the DY27 biosorption process was spontaneous and exothermic. FTIR and CSLM results confirmed that lignocellulose and proteins are involved in the biosorption process. Finally, a 0.1 M NaOH solution successfully eluted most of the DY27 dye from the DY27-loaded corncob biomass.

## References

[1] Dotto GL, Sanjay SK, Pinto LAA. Biosorption of organic dyes: Research opportunities and challenges In: Sharma SK, editor. Green chemistry for dyes removal from wastewater: research trends and applications. Scrivener Publishing LLC; 2015 pp. 295–329.

[2] Puentes-Cárdenas IJ, Chávez-Camarillo GM, Flores-Ortiz CM, Cristiani-Urbina MDC, Netzahuatl-Muñoz AR, Salcedo-Reyes JC, et al Adsorptive removal of acid blue 80 dye from aqueous solutions by Cu-TiO_2_. J Nanomater. 2016; 2016: ID 3542359.

[3] Yagub MT, Sen TK, Afroze S, Ang HM. Dye and its removal from aqueous solution by adsorption: A review. Adv Colloid Interface Sci. 2014; 209: 172–184. doi: 10.1016/j.cis.2014.04.002 24780401 10.1016/j.cis.2014.04.002

[4] Bharathi KS, Ramesh SPT. Fixed-bed column studies on biosorption of crystal violet from aqueous solution by *Citrullus lanatus rind* and *Cyperus rotundus*. Appl Water Sci. 2013; 3: 673–687.

[5] Du Y, Pei M, He Y, Yu F, Guo W, Wang L. Preparation, characterization and application of magnetic Fe_3_O_4_-CS for the adsorption of orange I from aqueous solutions. PLoS ONE. 2014; 9: e108647 doi: 10.1371/journal.pone.0108647 25271644 10.1371/journal.pone.0108647 PMC4182735

[6] Salleh MAM, Mahmoud DK, Karim WAWA, Idris A. Cationic and anionic dye adsorption by agricultural solid wastes: a comprehensive review. Desalination. 2011; 280: 1–13.

[7] Zaman A, Das P, Banerjee P. Biosorption of dye molecules In: Rathoure AK, Dhatwalia VK, editors. Toxicity and waste management using bioremediation. IGI Global; 2016 pp. 51–74.

[8] Devi P, Wahidullah S, Sheikh F, Pereira R, Narkhede N, Amonkar D, et al Biotransformation and detoxification of xylidine orange dye using immobilized cells of marine-derived *Lysinibacillus sphaericus* D3. Mar Drugs 2017; 15: 30. 10.3390/md15020030 PMC5334610 28208715

[9] Verma AK, Dash RR, Bhunia P. A review on chemical coagulation/flocculation technologies for removal of colour from textile wastewaters. J Environ Manage. 2012; 93: 154–168. doi: 10.1016/j.jenvman.2011.09.012 22054582 10.1016/j.jenvman.2011.09.012

[10] Apostol LC, Pereira L, Pereira R, Gavrilescu M, Alves MM. Biological decolorization of xanthene dyes by anaerobic granular biomass. Biodegradation. 2012; 23: 725–737. doi: 10.1007/s10532-012-9548-7 22437968 10.1007/s10532-012-9548-7

[11] Byberg R, Cobb J, Diez Martin L, Thompson RW, Camesano TA, Zahraa O, et al Comparison of photocatalytic degradation of dyes in relation to their structure. Environ Sci Pollut Res. 2013; 20: 3570–3581. 10.1007/s11356-013-1551-y 23423868

[12] Choi Y-S, Seo J-Y, Lee H, Yoo J, Jung J, Kim J-J, et al Decolorization and detoxification of wastewater containing industrial dyes by *Bjerkandera adusta* KUC9065. Water Air Soil Pollut. 2014; 225: 1801.

[13] Robinson T, McMullan G, Marchant R, Nigam P. Remediation of dyes in textile effluent: a critical review on current treatment technologies with a proposed alternative. Bioresour Technol. 2001; 77: 247–255. 11272011 10.1016/s0960-8524(00)00080-8

[14] El Haddad M, Regti A, Laamari MR, Slimani R, Mamouni R, El Antri S, et al Calcined mussel shells as a new and eco-friendly biosorbent to remove textile dyes from aqueous solutions. J Taiwan Inst Chem Eng. 2014; 45: 533–540.

[15] Guerrero-Coronilla I, Morales-Barrera L, Villegas-Garrido TL, Cristiani-Urbina E. Biosorption of amaranth dye from aqueous solution by roots, leaves, stems and the whole plant of *E*. *crassipes*. Environ Eng Manag J. 2014; 13: 1917–1926.

[16] Guerrero-Coronilla I, Morales-Barrera L, Cristiani-Urbina E. Kinetic, isotherm and thermodynamic studies of amaranth dye biosorption from aqueous solution onto water hyacinth leaves. J Environ Manage. 2015; 152: 99–108. doi: 10.1016/j.jenvman.2015.01.026 25617874 10.1016/j.jenvman.2015.01.026

[17] Aranda-García E, Cristiani-Urbina E. Effect of pH on hexavalent and total chromium removal from aqueous solutions by avocado shell using batch and continuous systems. Environ Sci Pollut Res. Forthcoming 2018 doi: 10.1007/s11356-017-0248-z 28963647 10.1007/s11356-017-0248-z

[18] Safa Y, Bhatti HN. Kinetic and thermodynamic modeling for the removal of Direct Red-31 and Direct Orange-26 dyes from aqueous solutions by rice husk. Desalination. 2011; 272: 313–322.

[19] Cao Q, Xie K-C, Lv Y-K, Bao W-R. Process effects on activated carbon with large specific surface area from corn cob. Bioresour Technol. 2006; 97: 110–115. doi: 10.1016/j.biortech.2005.02.026 16154508 10.1016/j.biortech.2005.02.026

[20] Garg UK, Kaur MP, Garg VK, Sud D. Removal of hexavalent chromium from aqueous solution by agricultural waste biomass. J Hazard Mater. 2007; 140: 60–68. doi: 10.1016/j.jhazmat.2006.06.056 16879918 10.1016/j.jhazmat.2006.06.056

[21] Córdoba JA, Salcedo E, Rodríguez R, Zamora JF, Manríquez R, Contreras H, et al Caracterización y valoración química del olote: degradación hidrotérmica bajo condiciones subcríticas. Rev Latinoamer Quím. 2013; 41: 171–184. Spanish.

[22] Tang S, Chen Y, Xie R, Jiang W, Jiang Y. Preparation of activated carbon from corn cob and its adsorption behavior on Cr(VI) removal. Water Sci Technol. 2016; 73: 2654–2661. doi: 10.2166/wst.2016.120 27232401 10.2166/wst.2016.120

[23] Vafakhah S, Bahrololoom ME, Bazarganlari R, Saeedikhani M. Removal of copper ions from electroplating effluent solutions with native corn cob and corn stalk and chemically modified corn stalk. J Environ Chem Eng. 2014; 2: 356–361.

[24] Ahmad T, Danish M, Rafatullah M, Ghazali A, Sulaiman O, Hashim R, et al The use of date palm as a potential adsorbent for wastewater treatment: a review. Environ Sci Pollut Res. 2012; 19: 1464–1484. 10.1007/s11356-011-0709-8 22207239

[25] Adeel S, Usman M, Haider W, Saeed M, Muneer M, Ali M. Dyeing of gamma irradiated cotton using direct yellow 12 and direct yellow 27: improvement in colour strength and fastness properties. Cellulose. 2015; 22: 2095–2105.

[26] Mahmud K, Islam MA, Mitsionis A, Albanis T, Vaimakis T. Adsorption of direct yellow 27 from water by poorly crystalline hydroxyapatite prepared via precipitation method. Desalin Water Treat. 2012; 41: 170–178.

[27] Sigma-Aldrich [Internet]. Direct Yellow 27 [cited 2017 Dec 29]. Available from: https://www.sigmaaldrich.com/catalog/product/sial/201871?lang=es&region=MX.

[28] Horwitz W, Latimer GW Jr. Official Methods of Analysis of AOAC International. 18^th^ ed. Maryland: Association of Official Analytical Chemists, AOAC International; 2005.

[29] Fiol N, Villaescusa I. Determination of sorbent point zero charge: usefulness in sorption studies. Environ Chem Lett. 2009; 7: 79–84.

[30] Lim LBL, Priyantha N, Mansor NHM. *Artocarpus altilis* (breadfruit) skin as a potential low-cost biosorbent for the removal of crystal violet dye: equilibrium, thermodynamics and kinetics studies. Environ Earth Sci. 2015; 73: 3239–3247.

[31] Netzahuatl-Muñoz AR, Cristiani-Urbina MDC, Cristiani-Urbina E. Chromium biosorption from Cr(VI) aqueous solutions by *Cupressus lusitanica* bark: Kinetics, equilibrium and thermodynamic studies. PLoS ONE. 2015; 10(9): e0137086 doi: 10.1371/journal.pone.0137086 26352933 10.1371/journal.pone.0137086 PMC4564179

[32] Vázquez-Palma DE, Netzahuatl-Muñoz AR, Pineda-Camacho G, Cristiani-Urbina E. Biosorptive removal of nickel(II) ions from aqueous solutions by Hass avocado (*Persea americana* Mill. var. Hass) shell as an effective and low-cost biosorbent. Fresen Environ Bull. 2017; 26: 3501–3513.

[33] Febrianto J, Kosasih AN, Sunarso J, Ju YH, Indraswati N, Ismadji S. Equilibrium and kinetic studies in adsorption of heavy metals using biosorbent: A summary of recent studies. J Hazard Mater. 2009; 162: 616–645. doi: 10.1016/j.jhazmat.2008.06.042 18656309 10.1016/j.jhazmat.2008.06.042

[34] Ho YS. Review of second-order models for adsorption systems. J Hazard Mater. 2006; B136: 681–689. 10.1016/j.jhazmat.2005.12.043 16460877

[35] Bharathi KS, Ramesh ST. Removal of dyes using agricultural waste as low-cost adsorbents: a review. Appl Water Sci. 2013; 3: 773–790.

[36] Vijayaraghavan K, Padmesh TVN, Palanivelu K, Velan M. Biosorption of nickel(II) ions onto *Sargassum wightii*: application of two-parameter and three-parameter isotherm models. J Hazard Mater. 2006; B133: 304–308. 10.1016/j.jhazmat.2005.10.016 16297540

[37] Kuo C-Y, Wu C-H, Wu J-Y. Adsorption of direct dyes from aqueous solutions by carbon nanotubes: determination of equilibrium, kinetics and thermodynamics parameters. J Colloid Interface Sci. 2008; 327: 308–315. doi: 10.1016/j.jcis.2008.08.038 18786679 10.1016/j.jcis.2008.08.038

[38] Leyva-Ramos R, Bernal-Jacome LA, Acosta-Rodriguez I. Adsorption of cadmium(II) from aqueous solution on natural and oxidized corncob. Sep Purif Technol. 2005; 45: 41–49.

[39] Saha P. Assessment on the removal of methylene blue dye using tamarind fruit shell as biosorbent. Water Air Soil Pollut. 2010; 213: 287–299.

[40] Schiewer S, Patil SB. Pectin-rich fruit wastes as biosorbents for heavy metal removal: equilibrium and kinetics. Bioresour Technol. 2008; 99: 1896–1903. doi: 10.1016/j.biortech.2007.03.060 17540559 10.1016/j.biortech.2007.03.060

[41] Tunç Ö, Tanaci H, Aksu Z. Potential use of cotton plant wastes for the removal of Remazol Black B reactive dye. J Hazard Mater. 2009; 163: 187–198. doi: 10.1016/j.jhazmat.2008.06.078 18675510 10.1016/j.jhazmat.2008.06.078

[42] Moyo M, Pakade VE, Modise SJ. Biosorption of lead(II) by chemically modified *Mangifera indica* seed shells: adsorbent preparation, characterization and performance assessment. Process Saf Environ Protect. 2017; 111: 40–51.

[43] Khataee AR, Vafaei F, Jannatkhah M. Biosorption of three textile dyes from contaminated water by filamentous green algal *Spirogyra* sp.: Kinetic, isotherm and thermodynamic studies. Int Biodeter Biodegr. 2013; 83: 33–40.

[44] Vijayaraghavan K, Yun Y-S. Biosorption of C.I. reactive black 5 from aqueous solution using acid-treated biomass of brown seaweed *Laminaria* sp. Dyes Pigments. 2008; 76: 726–732.

[45] Plazinski W, Rudzinski W, Plazinska A. Theoretical models of sorption kinetics including a surface reaction mechanism: A review. Adv Colloid Interface Sci. 2009; 152: 2–13. doi: 10.1016/j.cis.2009.07.009 19735907 10.1016/j.cis.2009.07.009

[46] Limousin G, Gaudet JP, Charlet L, Szenknect S, Barthès V, Krimissa M. Sorption isotherms: a review on physical bases, modeling and measurement. Appl Geochem. 2007; 22: 249–275.

[47] Aljeboree AM, Alshirifi AN, Alkaim AF. Kinetics and equilibrium study for the adsorption of textile dyes on coconut shell activated carbon. Arabian J Chem. 2017; 10: S3381–S3393.

[48] Dotto GL, Lima EC, Pinto LAA. Biosorption of food dyes onto *Spirulina platensis* nanoparticles: equilibrium isotherm and thermodynamic analysis. Bioresour Technol. 2012; 103: 123–130. doi: 10.1016/j.biortech.2011.10.038 22067438 10.1016/j.biortech.2011.10.038

[49] Dotto GL, Vieira MLG, Gonçalves JO, Pinto LAA. Remoção dos corantes azul brilhante, amarelo crepúsculo e amarelo tartrazina de soluções aquosas utilizando carvão ativado, terra ativada, terra diatomácea, quitina e quitosana: estudios de equilíbrio e termodinâmica. Quim Nova 2011; 34(7): 1193–1199. Portuguese.

[50] Barka N, Ouzaouit K, Abdennouri M, El Makhfouk M. Dried prickly pear (*Opuntia ficus indica*) cladodes as a low-cost and eco-friendly biosorbent for dyes removal from aqueous solutions. J Taiwan Inst Chem Eng. 2013; 44: 52–60.

[51] Cengiz S, Tanrikulu F, Aksu S. An alternative source of adsorbent for the removal of dyes from textile waters: *Posidonia oceanica* (L.). Chem Eng J. 2012; 189–190: 32–40.

[52] Foo KY, Hameed BH. Insights into the modeling of adsorption isotherm systems. Chem Eng J. 2010; 156: 2–10.

[53] Piccin JS, Vieira MLG, Gonçalves JO, Dotto GL, Pinto LAA. Adsorption of FD&C red No. 40 by chitosan: isotherm analysis. J Food Eng. 2009; 95: 16–20.

[54] Dotto GL, Moura JM, Cadaval TRS, Pinto LAA. Application of chitosan films for the removal of food dyes from aqueous solutions by adsorption. Chem Eng J. 2013; 214: 8–16.

[55] Hasan M, Ahmad AL, Hameed BH. Adsorption of reactive dye onto cross-linked chitosan/oil palm ash composite beads. Chem Eng J. 2008; 136: 164–172.

[56] Aksu Z, Isoglu IA. Use of agricultural waste sugar beet pulp for the removal of Gemazol turquoise blue-G reactive dye from aqueous solution. J Hazard Mater. 2006; B137: 418–430. 10.1016/j.jhazmat.2006.02.019 16603311

[57] Belhachemi M, Addoun F. Comparative adsorption isotherms and modeling of methylene blue onto activated carbons. Appl Water Sci. 2011; 1: 111–117.

[58] Hernández-Zamora M, Cristiani-Urbina E, Martínez-Jerónimo F, Perales-Vela HV, Ponce-Noyola T, Montes-Horcasitas MDC, et al Bioremoval of the azo dye Congo Red by the microalga *Chlorella vulgaris*. Environ Sci Pollut Res. 2015; 22(14): 10811–10823. 10.1007/s11356-015-4277-1 25772869

[59] Cheriti A, Talhi MF, Belboukhari N, Taleb S. Copper ions biosorption properties of biomass derived from Algerian Sahara plants In: Ning RY, editor. Expanding issues in desalination. Intech; 2011 pp. 319–338.

[60] Díaz-Muñoz LL, Bonilla-Petriciolet A, Reynel-Ávila HE, Mendoza-Castillo DI. Sorption of heavy metal ions from aqueous solution using acid-treated avocado kernel seeds and its FTIR-spectroscopy characterization. J Mol Liq. 2016; 215: 555–564.

[61] Calero M, Pérez A, Blázquez G, Ronda A, Martín-Lara MA. Characterization of chemically modified biosorbents from olive tree pruning for the biosorption of lead. Ecol Eng. 2013; 58: 344–354.

[62] Morán JI, Alvarez VA, Cyras VP, Vázquez A. Extraction of cellulose and preparation of nanocellulose from sisal fibers. Cellulose. 2008; 15: 149–159.

[63] Stuart B. Infrared spectroscopy: fundamentals and applications Chinchester: John Wiley and Sons; 2004.

[64] Kubo S, Kadla JF. Hydrogen bonding in lignin: a Fourier transform infrared model compound study. Biomacromolecules. 2005; 6: 2815–2821. doi: 10.1021/bm050288q 16153123 10.1021/bm050288q

[65] Ahmed F, Dewani R, Pervez MK, Mahboob SJ, Soomro SA. Non-destructive FT-IR analysis of mono azo dyes. Bulg Chem Commun. 2016; 48(1): 71–77.

[66] Liu R, Yu H, Huang Y. Structure and morphology of cellulose in wheat straw. Cellulose. 2005; 12: 25–34.

[67] Tondi G, Petutschnigg A. Middle infrared (ATR FT-MIR) characterization of industrial tannin extracts. Ind Crop Prod. 2015; 65: 422–428.

[68] Chan S-L, Tan YP, Abdullah AH, Ong S-T. Equilibrium, kinetic and thermodynamic studies of a new potential biosorbent for the removal of basic blue 3 and congo red dyes: Pineapple (*Ananas comosus*) plant stem. J Taiwan Inst Chem Eng. 2016; 61: 306–315.

[69] Coates J. Interpretation of infrared spectra, a practical approach In: Meyer RA, editor. Encyclopedia of analytical chemistry. Chinchester: Wiley; 2000 pp. 10815–10837.

[70] Wang S-L, Lee J-F. Reaction mechanism of hexavalent chromium with cellulose. Chem Eng J. 2011; 174: 289–295.

[71] Taşar Ş, Kaya F, Özer A. Biosorption of lead(II) ions from aqueous solution by peanut shells: equilibrium, thermodynamic and kinetic studies. J Environ Chem Eng. 2014; 2: 1018–1026.

[72] Lee M, Kim JH, Lee SH, Lee SH, Park CB. Biomimetic artificial photosynthesis by light-harvesting synthetic wood. ChemSusChem. 2011; 4: 581–586. doi: 10.1002/cssc.201100074 21506288 10.1002/cssc.201100074

[73] Pedrazzi R. Paper dyes In: Hunger K, editor. Industrial dyes: chemistry, properties, applications. Frankfurt: John Wiley & Sons; 2003 pp. 459–473.

[74] van de Velde F, Weinbreck F, Edelman MW, van der Linden E, Tromp RH. Visualization of biopolymer mixtures using confocal scanning laser microscopy (CSLM) and covalent labeling techniques. Colloid Surf B-Biointerfaces. 2003; 31: 159–168.

[75] Heidkamp GF, Heger L, Lehmann CHK, Dudziak D. Multi-parameter flow cytometry. Laboratory Journal—business web for users in science and industry. 31 5 2017 Available from: http://www.laboratory-journal.com/science/life-sciences-biotech/multi-parameter-flow-cytometry.

[76] De Micco V, Aronne G. Combined histochemistry and autofluorescence for identifying lignin distribution in cell walls. Biotech Histochem. 2007; 82(4–5): 209–216. doi: 10.1080/10520290701713981 18074267 10.1080/10520290701713981

[77] Rosas-Hernández Y. Extracción de compuestos celulósicos de bagazo de Agave angustifolia Haw obtenidos por organosolv asistido con microondas. M.Sc. Thesis, Centro de Desarrollo de Productos Bióticos, Instituto Politécnico Nacional. 2016. Available from: http://tesis.ipn.mx/bitstream/handle/123456789/20902/Tesis%20Yuliana%20Rosas%20Hernández.pdf?sequence=1&isAllowed=y. Spanish.

[78] Becker V, von Delius S, Bajbouj M, Karagianni A, Schmid RM, Meining A. Intravenous application of fluorescein for confocal laser scanning microscopy: evaluation of contrast dynamics and image quality with increasing injection-to-imaging time. Gastrointest Endosc. 2008; 68(2): 319–323. doi: 10.1016/j.gie.2008.01.033 18436217 10.1016/j.gie.2008.01.033

[79] Sigma-Aldrich [Internet]. Fluorescein [cited 2017 Dec 29]. Available from: https://www.sigmaaldrich.com/catalog/product/sigma/46955?lang=es&region=MX.

[80] Gip L, Abelin J. Differential staining of fungi in clinical specimens using fluorescent whitening agent (Blankophor). Mykosen. 1987; 30: 21–24. 2436047

[81] Ramos L, Mellado S, Ramadán S, Bulacio L, López C. Empleo de blanco de calcoflúor para el estudio de las especies de *Malassezia* por microscopía directa. Rev Arg Microbiol. 2006; 38: 4–8. Spanish. 16784125

[82] Sigma-Aldrich [Internet]. Calcofluor white stain [cited 2017 Dec 29]. Available from: https://www.sigmaaldrich.com/content/dam/sigma-aldrich/docs/Sigma-Calcofluorwhitestain.Aldrich/Datasheet/1/18909dat.pdf.

[83] Salvi NA, Chattopadhyay S. Biosorption of azo dyes by spent *Rhizopus arrhizus* biomass. Appl Water Sci. 2017; 7: 3041–3054.

